# Assessing the Multivariate Relationship between the Human Infant Intestinal Exfoliated Cell Transcriptome (Exfoliome) and Microbiome in Response to Diet

**DOI:** 10.3390/microorganisms8122032

**Published:** 2020-12-18

**Authors:** Kejun He, Sharon M. Donovan, Ivan V. Ivanov, Jennifer S. Goldsby, Laurie A. Davidson, Robert S. Chapkin

**Affiliations:** 1 Center for Applied Statistics and Institute of Statistics and Big Data, Renmin University of China, Beijing 100872, China; kejunhe@ruc.edu.cn; 2 Department of Food Science and Human Nutrition, University of Illinois, Urbana, IL 61801, USA; sdonovan@illinois.edu; 3 Veterinary Physiology and Pharmacology, Texas A&M University, College Station, TX 77843, USA; ivanov@tamu.edu; 4 Program in Integrative Nutrition and Complex Diseases, Texas A&M University, College Station, TX 77843, USA; jsgoldsby@tamu.edu (J.S.G.); l-davidson@tamu.edu (L.A.D.); 5 Department of Nutrition, Biochemistry & Biophysics, Texas A&M University, College Station, TX 77843, USA

**Keywords:** microbiota, exfoliome, infant, sparse canonical correlation analysis, sparse principal components analysis, breast milk

## Abstract

Gut microbiota and the host exist in a mutualistic relationship, with the functional composition of the microbiota strongly influencing the health and well-being of the host. In addition to the standard differential expression analysis of host genes to assess the complex cross-talk between environment (diet), microbiome, and host intestinal physiology, data-driven integrative approaches are needed to identify potential biomarkers of both host genes and microbial communities that characterize these interactions. Our findings demonstrate that the complementary application of univariate differential gene expression analysis and multivariate approaches such as sparse Canonical Correlation Analysis (sCCA) and sparse Principal Components Analysis (sPCA) can be used to integrate data from both the healthy infant gut microbial community and host transcriptome (exfoliome) using stool derived exfoliated cells shed from the gut. These approaches reveal host genes and microbial functional categories related to the feeding phenotype of the infants. Our findings also confirm that combinatorial noninvasive -omic approaches provide an integrative genomics-based perspective of neonatal host-gut microbiome interactions.

## 1. Introduction

Early microbial colonization in infants is critically important for directing neonatal intestinal and immune development and is especially attractive for studying the development of human-commensal interactions [1,2]. Hence, it is imperative to understand the adaptive responses of the neonatal gut to diet, the intestinal microbiome, and microbial metabolites. However, access to tissue biopsies from healthy human infants is impossible, therefore our group has previously established and validated a methodology using stool derived exfoliated cells from the gut to interrogate the responses of the neonatal intestinal global transcriptome, i.e., exfoliome, to dietary substrates in the early neonatal period [3,4]. Each day, ~10 billion cells are exfoliated from the intestinal lining as part of normal epithelial cell turnover [5]. Exfoliated cells undergo anoikis and autophagy, rather than apoptosis [6,7], promoting cell survival for a limited period of time [8]. Exfoliated cells in stool have been used in children with inflammatory bowel disease [9] and in stool [8] and gastric aspirates of preterm infants [10] to study cellular and molecular markers. 

Using exfoliated cells, we applied novel transcriptome-based methods to identify the best single-gene, two-to-three gene combinations (biomarkers) to distinguish between dietary treatments, e.g., fingerprint/classify breast- (BF) vs. formula-fed (FF) term infants [3]. The best single gene classifier was endothelial PAS domain-containing protein (EPAS1; also known as hypoxia-inducible factor-2alpha), which also performed well in multivariate sets of 2 and 3-gene combinations [3]. Comparing the exfoliome of term and preterm infants revealed the functional immaturity of signaling pathways controlling cell proliferation and long-chain polyunsaturated fatty acid synthesis in preterm infants, coupled with up-regulated immune and inflammatory gene pathways in preterm infants [4]. In addition, we have recently demonstrated that the non-invasive exfoliated transcriptome reflects the tissue-level transcriptome in a mouse model of non-steroidal anti-inflammatory drug enteropathy [11]. These novel findings have provided insight into the global patterns of gene expression that vary in exfoliated epithelial cells of term and preterm infants. 

As part of an ever-expanding effort to generate predictive genomic network data, our interdisciplinary team has pioneered molecular and systems biology methodologies for simultaneously monitoring host gastrointestinal exfoliome gene expression and the gut microbiota [12]. In term infants, microbiome composition was characterized by 16S rRNA [13] and metagenome shotgun sequencing [14] of BF and FF neonates. At the phyla level, the relative abundance of Actinobacteria was not significantly different between BF (60%) and FF (54%) infants. In contrast, the relative abundance of Bacteroidetes was significantly greater (21% vs. 0.03%) and Firmicutes was significantly lower (13% vs 37%) in BF vs. FF infants, respectively [12]. Metagenomic analysis of the same samples demonstrated significant differences in the abundance of virulence SEEDLevel2 microbial categories [14]. Thus, we used sub-dimensional Canonical Correlation Analysis (CCA) to detect the correlative structure between the virulence SEEDLevel2 microbial categories and a prior knowledge list containing immunology and defense related host genes [14]. However, due to the sub-dimensional application of classical CCA, the small number of samples, the large number of measured variables, and the inherent sparsity present in the data, analyses were limited to gene sets of size 3. 

Based on these initial findings, the goal of this study is to examine the multivariate structure of host intestinal exfoliome expressed genes related to immunology [14], microbial fermentation [15,16] and barrier function [3] in combination with microbiome-derived DNA sequences in three-month-old exclusively BF and FF infants. Our initial analysis of the microbial fermentation products (volatile fatty acids; VFA) in the same group of infants provides rationale for the application of variations of classical CCA and principal component analysis (PCA), i.e., sCCA and sPCA, to the data sets described and published in our previous work [14]. 

Our proof-of-principle approach provides novel insight into the structure of each data type (microbial and host exfoliome) in isolation and suggests potential host-microbiome interactions following the introduction of dietary substrates in the early neonatal period. This approach extends our previous work [14] and incorporates prior knowledge in the data analyses. Furthermore, we compare the performance of two recently developed analytical methods, sCCA and sPCA [17,18], in relation to the same data set [14]. This comparison provides new insight into the complex gut-microbiota system. We also compare results obtained by the application of sPCA and sCCA to the more “traditional” univariate approach of testing for differentially expressed (DE) host genes to illustrate that sCCA and sPCA have the potential to uncover additional multivariate structures in the same data set.

## 2. Materials and Methods 

### 2.1. Human Subjects

The source of the human samples analyzed herein was previously described [3]. Briefly, freshly voided stool samples were obtained from three-month-old, healthy, vaginally-delivered, exclusively BF or FF infants for isolation of exfoliated cell mRNA and microbiome RNA and DNA. Samples were immediately placed in denaturation solution to preserve sample quality [3]. The human subjects protocol was approved by the Institutional Review Boards of the University of Illinois, Urbana-Champaign and Texas A&M University. Informed consent was obtained from parents prior to participation in the study.

### 2.2. Isolation and Analysis of Stool Microbial DNA and Host PolyA^+^ mRNA

Methods for isolating and quantitating stool microbial DNA and host polyA^+^ mRNA were previously described [4,11]. For host exfoliome analyses, samples were processed in strict accordance to the CodeLink™ Host Gene Expression Assay manual (Applied Microarray, Tempe, AZ, USA) and analyzed using the Human Whole Genome Expression Bioarray, as we have previously described [19]. Metagenomic sequencing of microbial DNA was previously described in Schwartz et al. [14]. Metagenome functions were assigned using SEED functional categories [20]. The general workflow for the types of data preprocessing and analyses is described in Figure S1.

### 2.3. Data Normalization, Transformation and Prior Knowledge Lists

To assess the impact of diet on microbiota functional characteristics, microbial data were aligned, as previously described in [14] using MG-RASTv2 against the second level SEED subsystem database [21]. Subsequently, normalization and taxonomic classifications were performed as described in [22,23]. After aligning to the second level SEED subsystem and applying quality control filtering and normalization, 115 SEEDLevel2 categories (Table S1) remained available for the sCCA and sPCA analyses. Microarray raw gene expression data was logarithmic transformed, and quantile normalized as in [24]. The median value for the probes with the same ID was utilized to represent the signal for those probes because the median value has been shown to be more robust in quantile normalization procedures [25]. When performing either sCCA or sPCA analyses, normalized expression values for three prior knowledge lists of host genes related to short chain fatty acids (SCFA) (72 genes) (Table S2), immunology (811 genes) (Table S3), and barrier function (52 genes) (Table S4) were utilized, as previously described [3,14].

### 2.4. Differential Gene Expression Analysis

Normalized host gene expression values were used to test for significant differences in expression (DE) when comparing BF vs. FF infants [26]. All probes on the microarray chip that passed the preprocessing steps were used for the DE analysis.

### 2.5. Quantification of Fecal Volatile Fatty Acids

Short chain (acetate, propionate and butyrate) and branched chain (isobutyrate, isovalerate, and valerate) fatty acid concentrations were analyzed by gas chromatography, as previously described [27] and are expressed per gram feces on a dry matter basis. 

### 2.6. Gut Metagenome and Host Transcriptome Multivariate Analyses and Data Integration

We elected to compare the performance of two multivariate analyses methods, sPCA and sCCA, on both synthetically generated data and on our infant dataset. sPCA and sCCA are recent modifications of the classical Principal Component Analysis (PCA) and Canonical Correlation Analysis (CCA) [17,28]. PCA is a dimension-reduction method for analyzing a set of multivariate data and has a wide range of applications throughout science and engineering. PCA aims at replacing the original variables by a small number of uncorrelated features through linear combinations of the original variables, so that the new ones may explain the most of the variation in the data. Those new uncorrelated variables are called the principal components (PCs) and the direction vectors of the original variables are also known as loadings. In comparison, CCA is a method of correlating linear relationships between two sets of multidimensional variables [28]. Intuitively speaking, CCA can be seen as the solution of the problem of finding basis vectors for two sets of variables such that the correlation between the projections of the variables onto these basis vectors are mutually maximized. The main difference between CCA and PCA is that CCA is closely related to mutual information, while PCA deals with the marginal information alone. Additional technical information about PCA, CCA, sPCA, and sCCA is presented in Appendix A.1. Because the dimensionality of the measured variables in our data is much larger than the number of the available biological samples, we used sparse versions of PCA and CCA, sPCA and sCCA, respectively [17,18]. sCCA was utilized to assess the multivariate relationships between the gut microbial metagenomic and host transcriptomic data [18]. The results of a simulation study (Appendix A.2) were used to inform our selection of methods for integrative data analysis. These findings are consistent with the view that sCCA offers a viable alternative to the sub-dimensional application of CCA [14] in situations where the number of samples is much smaller than the number of measured variables. For example, it allows for a single optimization procedure over the space of all of the variables and does not suffer from the ad hoc threshold selection procedures of the sub-dimensional CCA. In the cases where the application of sCCA did not reveal any mutual correlative structure between the two data types, we applied sPCA to visualize any potential grouping of the samples. sPCA relates to sCCA in the same way the traditional Principal Component Analysis (PCA) relates to CCA [17,28,29,30]. Another important consideration for utilizing the sparse approach (sPCA or sCCA) was the performance of the simpler of the two approaches (sPCA) on each particular data set, i.e., when sPCA provided a separation between the samples from the two feeding phenotypes. This would warrant a closer look at the sCCA results and the composition of the corresponding principal components. 

### 2.7. Data Deposition

The metagenome sequence data have been deposited in the European Bioinformatics Institute’s Short Read Archive (ERP001038). The human exfoliome data have been deposited in the NCBI Gene Expression Omnibus (GSE31075).

## 3. Results

### 3.1. Fecal Volatile Fatty Acid Concentrations

Analysis of VFA concentrations in feces demonstrated differences in both short-chain fatty acids (SCFA) and branched-chain fatty acids (BCFA) between BF and FF infants (Table 1). Alterations in these microbial metabolites could be explained by differences in fermentable substrates in human milk vs. formula, microbial populations or diet-induced differences in the expression of host genes associated with host SCFA uptake and metabolism. Based on these differences, we applied both univariate analysis of differential gene expression and multivariate sPCA and sCCA to genes associated with SCFA receptor signaling. Interestingly, the univariate testing of the expression of this class of genes was not associated with FDR corrected *p*-values levels of 0.05.

### 3.2. Anatomic Origin of Exfoliated Intestinal Epithelial Cells

**Figure 1:**
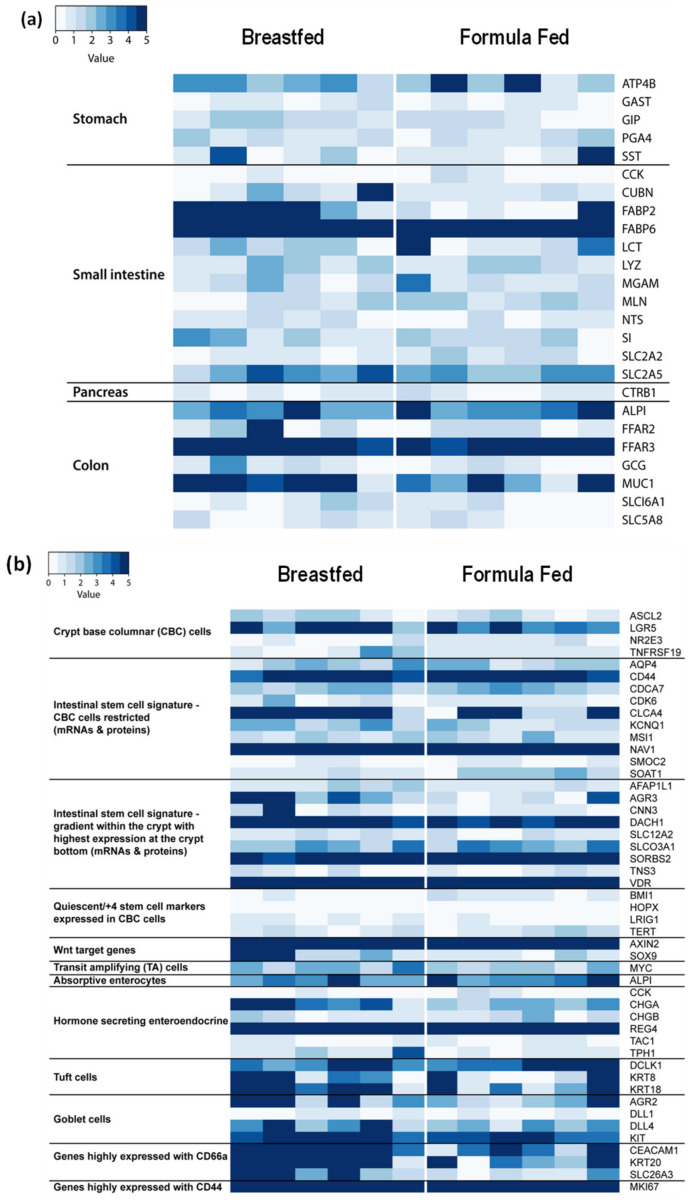
The exfoliome signatures related to anatomic locations and cell types. Heat map of the relative expression of genes expressed at specific anatomic locations (stomach, small intestine, pancreas, and colon) (**a**). Heat map of genes associated with specific cell types (**b**). Highly expressed genes are colored in dark blue.

The composition of exfoliated intestinal epithelial cells may directly contribute to alterations in gene expression, thus the anatomic origin of the host gene expression signature derived from the exfoliated cells was determined. The relative expression of genes previously identified and expressed predominantly in specific anatomic locations (i.e., stomach, pancreas, small intestine, and colon) were quantified (Figure 1a). Signatures arising from the stomach, small intestine and colon were detected. The intestinal mucosa is comprised of numerous cell types (stem cells, crypt cells, enteroendocrine cells, goblet cells, Paneth cells, and immune infiltrating cells), therefore, we also evaluated the expression of marker genes expressed either solely, or at least highly enriched, in a specific cell type [11]. Analysis of the exfoliome revealed the expression of marker genes typically associated with a wide array of intestinal epithelial cell types, e.g., stem cells, crypt base columnar, enteroendocrine, goblet, and tuft cells (Figure 1b). In addition, genes associated with innate and adaptive immune response cellular functions (CD44 and CD66a) were highly expressed. 

### 3.3. Data Structure and Interactions between the Host Transcriptome and Gut Microbiome in Breast- and Formula-Fed Neonates

Since the application of either CCA or PCA to multi-omic data in situations where the number of samples is much smaller than the number of measured variables presents challenges related to the mathematical operations on the data matrixes, we used optimized sparse versions of CCA and PCA: sCCA and sPCA respectively [17,18]. As a result, we identified linear combinations of gut microbial genes and genes from the intestinal host exfoliated cell transcriptome that discriminate between the two infant feeding phenotypes. It is important to note that sCCA offers a viable alternative to the sub-dimensional application of CCA. Moreover, it allows for integration of data from both microbiome and host exfoliome, which is not possible when applying sPCA [18] on single type data or performing classical univariate statistical testing for differential gene expression. Since sCCA may fail to provide meaningful results in certain instances where the detectable mutually correlative structure between the two types of data is lacking, we also applied sPCA to each data type separately to explore the relevant multivariate data structure as revealed by the composition of the corresponding principal components. Based on our preliminary findings examining the effects of diet on neonatal intestinal gene expression [14] and the results of our simulation study regarding the performance of sCCA, sPCA, and sub-dimensional CCA in our comparative simulation study (Appendix A), we initially queried the composition of first and second canonical sCCA components. Specifically, three groups of genes generated from previous findings [3,4] and a review of the literature were each subjected to sCCA together with microbial SEEDLevel2 data (Table S1). The three groups were comprised of 72 SCFA receptor signaling genes (Table S2), 811 host immunity and defense genes (Table S3), and 52 intestinal barrier function genes (Table S4). In each case, the number of genes in the respective lists were far larger than the infant sample size of 12 and the classical CCA failed to provide solutions (data not shown). Therefore, sCCA was subsequently used to detect the mutually correlative structure present in the combined host gene expression and microbial SEEDLevel2 data sets. We also performed sPCA on the above-mentioned gene lists for the purpose of discovering any additional structure present in our data. As shown in Figure S2, the application of sPCA to the data from the SEEDLevel2 microbial categories did not produce separation between the two feeding phenotypes. 

#### 3.3.1. SCFA Signaling Genes and Microbial SEEDLevel2 Categories

**Figure 2:**
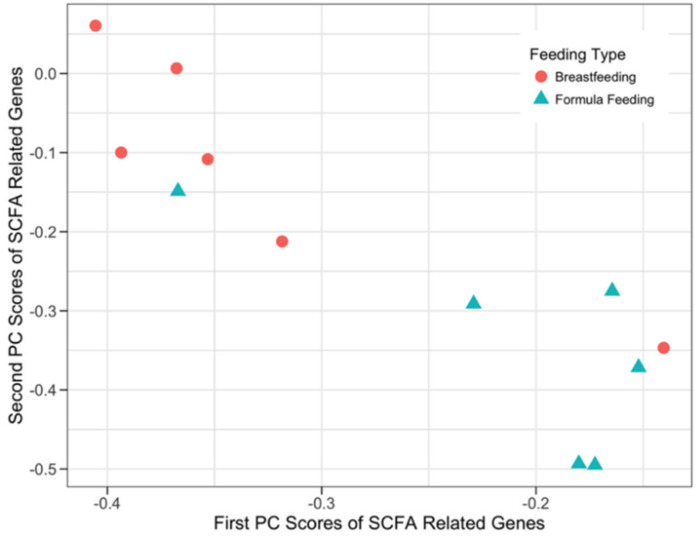
Sparse Principal Component Analysis (sPCA) results for the short-chain fatty acids (SCFA) receptor related host genes. The x-axis and the y-axis represent the first and the second principal components obtained after the sPCA application to the normalized microarray expression of SCFA receptor-related host genes, respectively. A total of 33 out of 72 SCFA receptor-related genes participated in the first two principal components of this sPCA analysis.

Both sPCA and sCCA were applied to the microbial SEEDLevel2 categories (Table S1) and the 72 genes associated with SCFA receptor signaling (Table S2) to evaluate their ability to separate the two infant feeding phenotypes (BF and FF). The application of sPCA separated the two feeding phenotypes (Figure 2) and identified 27 genes participating in the first component, 22 forming the second component, and 16 genes being represented in both components (Table S5). Interestingly, GPR41 (SCFA receptor-related gene) participated in the first component, whereas GPR43 SCFA receptor-related gene participated in the second component. Notably, none of the 72 SFCA receptor-related genes exhibited a significant difference (FDR-corrected *p*-value < 0.05) in expression between the two infant feeding phenotypes. In addition, sCCA analysis did not detect any correlative structure between SFCA host genes and data from SEEDLevel2 microbial categories (Figure S3). 

#### 3.3.2. Host Immunology and Defense Genes and Microbial SEEDLevel2 Categories

**Figure 3:**
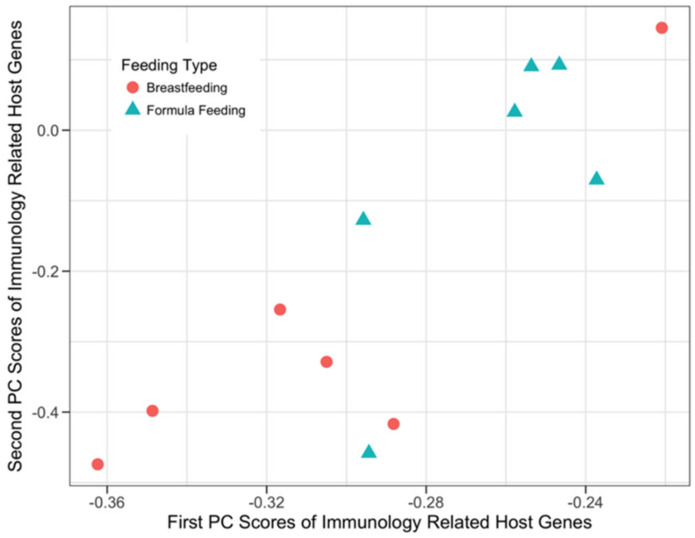
sPCA of immunology and defense related host genes. The x-axis and the y-axis represent the first and the second component scores from the normalized microarray expression of the immunology and defense related host genes, respectively. A total of 76 out of 811 immunology and defense-related genes participated in the first two principal components of this sPCA analysis.

Both sPCA and sCCA were applied to the prior knowledge list of 811 host immunology and defense-related genes (Table S3) and the microbial SEEDLevel2 categories (Table S1) to evaluate their ability to separate the two infant feeding phenotypes (BF and FF). Application of sPCA (Figure 3) resulted in separation between the two feeding phenotypes and identified 45 and 39 genes with non-zero loadings in the first and second principal components, respectively (Table S6), with eight genes being represented in both components. 

**Figure 4:**
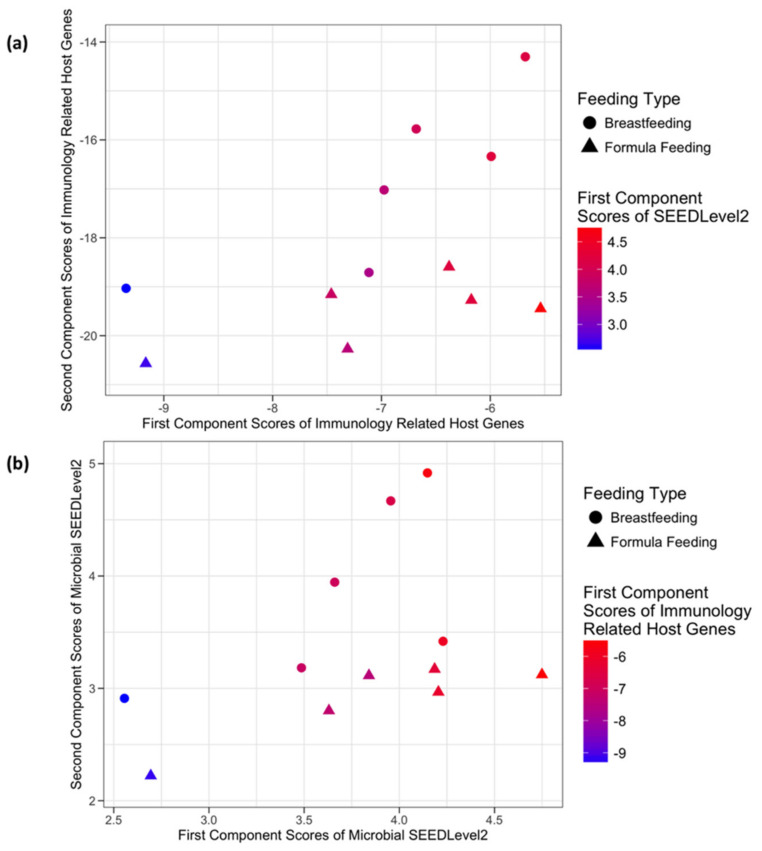
Sparse Canonical Correlation Analysis (sCCA) of immunology and defense related host genes and the microbial communities at SEEDLevel2. In panel (**a**), the x-axis and the y-axis represent the first and the second component scores from the normalized microarray expression of immunology and defense related host genes, respectively. The list of genes forming these two components is presented in Table S7a. Coloring of the sample points represents the first component scores from the microbial communities from the SEEDLevel2 subsystem. In panel (**b**), the x-axis and the y-axis represent the first and the second component scores from the normalized SEEDLevel2 subsystem of microbial communities, respectively. The list of SEEDLevel2 microbial categories forming these two components is presented in Table S7b. Coloring of the sample points represents the first component scores from the normalized microarray expression of the immunology and defense related host genes.

The application of sCCA to immunology and defense related host genes and microbial communities at SEEDLevel2 identified 15 genes participating in the first component and 12 forming the second component with no genes shared by the two components. The combined host expression and microbial data structure from the perspective of the host genes in relation to the microbial SEEDLevel2 categories is shown in Figure 4a and Table S7a. Similarly, Figure 4b describes the data structure from the perspective of the microbial SEEDLevel2 categories in relation to their putative interactions with immunity and defense-related host genes. The SEEDLevel2 categories forming the respective first two components are shown in Table S7b. 

#### 3.3.3. Host Barrier Function-Related Genes and Microbial SEEDLevel2 Categories

**Figure 5:**
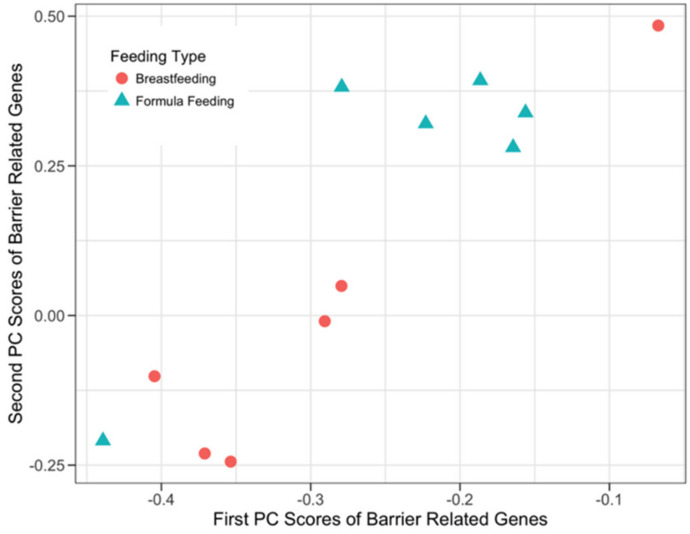
sPCA plot for barrier-related host genes. The x-axis and the y-axis represent the first and the second principal components obtained after the sPCA application to the normalized barrier-related host genes. A total of 24 out of 52 host barrier function-related genes participated in the first two principal components.

**Figure 6:**
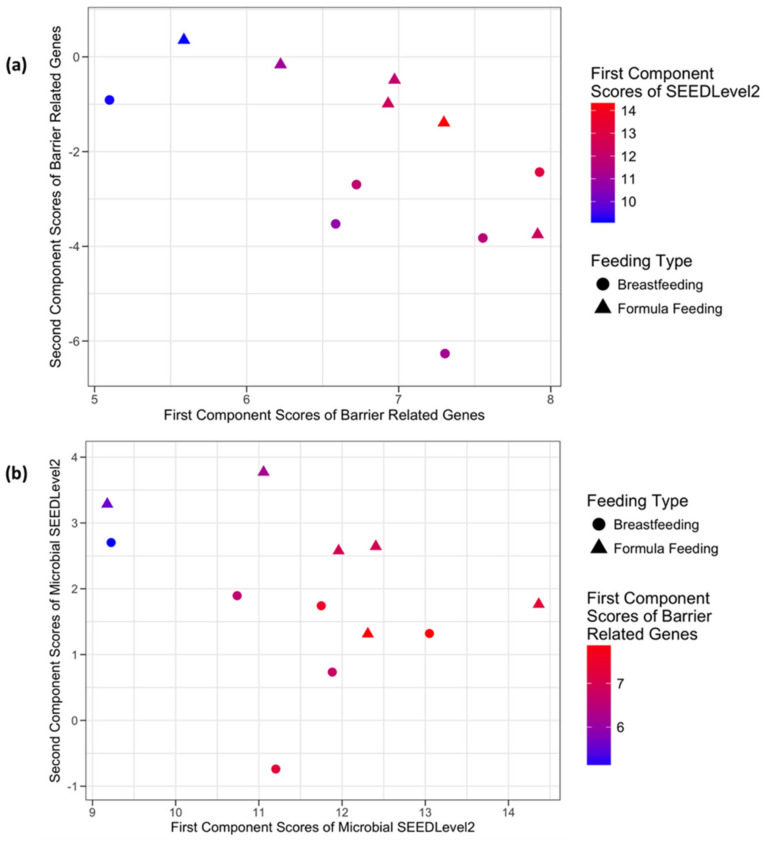
sCCA plots for barrier related host genes and the microbial communities at SEEDLevel2. In panel (**a**), the x-axis and the y-axis represent the first and the second component scores from the normalized microarray expression of the barrier related host genes, respectively. The list of genes forming these two components is described in Table S9a. Coloring of the sample points represents the first component scores from the microbial communities from the SEEDLevel2 subsystem. In panel (**b**), the x-axis and the y-axis represent the first and the second component scores from the normalized SEEDLevel2 subsystem of microbial communities, respectively. The list of genes forming these two components is described in Table S9a. Coloring of the sample points represents the first component scores from the normalized microarray expression of the barrier related host genes.

The data structure and putative relationship between host barrier function-related genes and microbial SEEDLevel2 metabolic function categories was queried using a list of 52 barrier function-related genes (Table S4). sPCA (Figure 5) analysis showed separation between the two feeding phenotypes and identified 18 and 16 genes with non-zero loadings in the first and second principal components, respectively (Table S8), with 10 genes being represented in both components. In comparison, sCCA identified four genes participating in the first component and two forming the second component (Table S9a), with no shared genes between the two components. The combined host expression and microbial data structure from the perspective of mucosal barrier-related host genes in relation to their putative interactions with the microbial SEEDLevel2 categories is shown in Figure 6a. Similarly, Figure 6b describes the data structure from the perspective of the categories detectable at microbial SEEDLevel2 in relation to the mucosal barrier-related host genes. In this context, sCCA identified 12 categories forming the first and 11 forming the second component (Table S9b). 

### 3.4. Differential Gene Expression

We also performed complementary differential expression testing for genes represented by the three prior knowledge gene lists. Table 2 lists the relative expression level for eight of the most highly significantly differentially expressed (DE) genes from the three gene category lists (SCFA, immunity and defense, or mucosal barrier function) in BF vs. FF infants. Of these, NR3C1, LTBP4, DEFB118, and CTNND1 exhibited multivariate relationships to microbiota SEEDLevel2 characteristics, as reflected in the sCCA or sPCA components. It is noteworthy, that this univariate analysis does not detect highly significant differences in gene expression as reported by the respective FDR-corrected *p*-values/*q*-values (Table 2). This observation underscores the importance of multivariate approaches (sPCA, sCCA) that have the potential to detect additional data structure and integrate data from different modalities (sCCA).

## 4. Discussion

Our data analyses used both univariate (testing for DE of genes) and multivariate approaches (sPCA and sCCA) to assess host-microbe crosstalk in healthy human infants. Importantly, testing for DE genes did not detect many strongly DE genes after the appropriate FDR correction of the raw p-values (Table 2). These findings emphasize the importance of multivariate approaches as described by the results of the application of sCCA and sPCA. In most cases (Figure 3, Figure 4, Figure 5 and Figure 6) where multivariate methods were deployed, separation between the two feeding infant phenotypes (BF and FF) was detected. This suggests that the respective composition of the principal components contains genes or SEEDLevel2 microbial categories that could provide deeper insight into the structure of data and putative relationships between host gut epithelium and microbial communities then a “traditional” univariate DE gene analysis. Interestingly, the application of sCCA to the combination of SFCA host genes and the SEEDLevel2 microbial categories did not reveal any mutually correlative data structure. In contrast, sPCA application to the SCFA host genes detected a data structure consistent with the separation of the two infant phenotypes (Figure 2). 

### 4.1. Anatomical Source of Exfoliated Cells

We have previously reported that the exfoliated transcriptome reflects the tissue-level transcriptome in a mouse model of NSAID enteropathy [11]. Although we did not attempt to identify the precise sources of cells, marker genes representing various anatomical regions and cell types revealed that the signature in the neonatal exfoliome was derived from both the stomach, small intestine and colon with virtually no signature coming from the pancreas (Figure 1a). Many of the cell types associated with the intestinal mucosa, e.g., stem cells, crypt cells, enteroendocrine cells, goblet cells, Paneth cells, and immune infiltrating cells, were present in the exfoliome (Figure 1b), suggesting the contribution of a variety of mucosal cell types. These findings corroborate and extend previous observations made in adult humans [31].

### 4.2. Data Structure Detected by sPCA

While sPCA does not aim to detect potential interactions between host genes and the microbial SEEDLevel2 categories, it can detect structures in each individual data type that might not be related to mutual correlative relationships between these two data types. Therefore, we applied sPCA to evaluate the general structure of each one of the three prior knowledge gene lists (Tables S2–S4) and the SEEDLevel2 microbial categories (Table S1). sPCA separated the two feeding infant phenotypes when each individual prior knowledge gene list was considered (Figure 2, Figure 3, Figure 5 and Tables S5–S7). However, no such separation was present for the SEEDLevel2 microbial categories (Figure S2). 

### 4.3. Correlative Data Structure Detected by sCCA

CCA is a statistical method for exploring the correlative relationships between two multivariate sets of variables [28]. The canonical correlation coefficient measures the strength of association between the canonical variates formed by appropriate linear combinations of the original variables. These linear combinations are the result of an optimization procedure that maximizes the correlation between the respective pairs of canonical variates. However, when the number of measured variables, e.g., several hundred or thousands, is much greater than the number of observations/samples, the classical CCA cannot be applied directly. One potential remedy in such situations is to apply CCA in a sub-dimensional manner, an approach previously adopted by our group [14]. Although intuitive in nature, the sub-dimensional CCA does not provide a rigorous statistical approach, because of the arbitrary imposed thresholds. Moreover, the exhaustive search over the space of all combinations of variables of a certain size is computationally expensive and only modest (three to four variables at a time) sub-dimensional searches can be performed when the total number of variables in one of the data sets is in order of tens of thousands. To obviate these constraints, we integrated microbial DNA and eukaryotic stool mRNA sequencing data (exfoliome) from the healthy infant gut utilizing a sCCA approach. This process allowed us to identify previously undetected molecular signatures whereby environmental factors (diet) potentially influence the cross-talk (mutualism) between the gut microbiota and the host. 

### 4.4. Description of Genes Identified by Multivariate Analysis

Genes identified by the multivariate analysis include free fatty acid receptor-2 (FFAR2 or GPR43) and FFAR3 (GPR41) (Table S5). This is noteworthy, because SCFAs act as signal transduction molecules via G-protein coupled receptors [32]. The two receptors are coupled to inositol 1,4,5-trisphosphate formation, intracellular calcium release, ERK1/2 activation and inhibition of cAMP accumulation [32], and are expressed in the gut, pancreatic β-cells, adipose, immune and neural cells [32]. These receptors differ in their affinity for SCFAs, tissue distribution, and physiological roles. Acetate preferentially activates FFAR2, propionate mainly activates FFAR3, and butyrate equally activates both FFAR2 and FFAR3 [33]. 

We have previously demonstrated that virulence characteristics of the microbiome exhibit differential sensitivity to breast milk as compared to formula [14]. Therefore, we also focused our transcriptomic analysis on host genes associated with host immunity/defense and those associated with intestinal barrier function. By adapting sCCA outcomes, we identified a subset of 27 immunity and defense related genes and six barrier function-related genes that exhibited evidence of a multivariate relationship with microbiome SEEDLevel2 categories. It is noteworthy that intestinal barrier function is regulated in part by immunological stimuli, particularly proinflammatory cytokines [34]. Of the 27 immunity and defense genes, 20 had a higher mean expression in FF than BF infants, suggesting that diet modulated the interaction between the microbial virulence characteristics and host gene expression. Three cytokines, IL-17, IL-22 and interferon-α, all had lower mean expression in the BF than FF exfoliome. IL-17 and IL-22 are both secreted by Th17 cells, a lineage of effector CD4 T cells [35]. In the intestinal mucosa, IL-17 and IL-22 expression is induced by microbial amyloids binding to toll-like receptors [36]. Amyloid fibrils are produced by members of the phyla Firmicutes, Bacteroidetes, and Proteobacteria [36]. IL-17 is a pro-inflammatory cytokine that orchestrates protection against infectious pathogens by enhancing the epithelial release of antimicrobial peptides, granulopoiesis, and neutrophil accumulation in peripheral tissues [37]. IL-17 also induces claudin 1 and claudin 2, that are involved in the formation of tight junctions between cells in the human gut epithelium, thus ensuring intestinal integrity. In addition, Claudin 1 (*CLDN1*) was identified by sCCA as a barrier function gene associated with microbiome virulence characteristics. IL-22 is a homeostatic cytokine preserving the integrity of boundary organs and tissues, and is only occasionally proinflammatory [37]. IL-17 and IL-22 also promote the release of β-defensin-2 and β-defensin-3, which contribute to the immune response against bacterial, fungal, and viral infections [38]. Unexpectedly, given the lower expression of IL-17 and 22 in BF vs. FF infants, β-defensin was up-regulated in BF vs. FF infants. Not much is known about the function of this β-defensin, but it is a ligand for the CC-chemokine receptor CCR2, as is β-defensin-2 [39]. Additional potential crosstalk between the genes identified by sCCA is suggested by the fact that transforming growth factor beta (TGF-β) is needed for optimal expression of IL-22 induced by IL-1 β [35]. We observed a higher expression of LTBP4 in BF infants, which would inhibit the activation of TGF-β and, potentially, IL-22 expression. 

Genes related to T- and B-cell function were identified by our analyses. For example, the transcription factor CEBPB (CCAAT/enhancer-binding protein beta), which reduces proliferation and promotes expression of differentiation-related genes in T-cells [40], was up-regulated in BF infants. Two genes that were down-regulated in BF vs. FF infants were associated with T-cell function. CDE3, which is part of the T-cell receptor CD3 complex on T-cell membranes, plays a role in adaptive immune response [41], and PTPN22 (protein-tyrosine phosphatase 22), a non-receptor protein-tyrosine phosphatase represses signaling through the T cell receptor [42]. Two additional genes were associated with B-cell function. *CLCF1* (Cardiotrophin-like cytokine factor 1), which stimulates B-cell proliferation and Ig production [43] and CD22, which is a member of the Siglec family that binds α2,6-linked sialic acids. CD22 inhibits B-cell receptor induced signaling and has a role in preventing autoimmunity [44]. Taken together, these gene expression profiles suggest a lower proinflammatory tone in the intestinal exfoliome of BF vs. FF infants, in which diet mediates the interactions between the microbial genes and host immune responses. 

With respect to intestinal barrier function, fewer genes were identified, but included claudin 1 (CLDN1) and claudin-4 (CLDN4). Claudins are a family of small transmembrane proteins which, along with occludin, are the most important components of the tight junctions [34]. Claudin-4 was expressed at a higher level in BF, whereas claudin-1 was expressed at a lower level in BF compared to FF. A similar relationship between the two proteins was observed in the non-lesional skin of patients with atopic dermatitis, in which CLDN1 was down-regulated and CLDN4 was up-regulated [45]. Genes for two forms of actin were also identified, G1 (*ACTG1*) and A4 (*ACTA4*), which were up- and down-regulated in BF vs. FF, respectively. The G actins assemble into polarized filaments that form networks impacting the cytoskeleton and generate force to support internal cell motility [46]. 

Chemokine ligand 2 (CXCL2) expression was higher in BF infants, which seems counterintuitive to the general lower expression of inflammatory markers. This barrier function gene, also designated macrophage inflammatory protein 2-alpha, has been associated with inflammatory diseases and is chemotactic for neutrophils [47]. Neutrophils are important for killing invading pathogens, but this process produces reactive oxygen species and releases proteases that can damage tissue and reduce barrier function [48]. However, a recent study demonstrated that exposing a fecal intestinal epithelial cell line to human milk up-regulated the expression of four chemokine genes, including CXCL2 [49]. Interestingly, CXCL2 along with IL-6 and CXCL10 were linked to the GO term “Response to Molecular of Bacterial Origin” (GO:0002237), suggesting a potential link to microbial components, which in this context would be the milk microbiome [49].

Of the 27 genes identified by sCCA, NR3C1, LTBP4, and CTNND1 showed the greatest difference in expression between BF and FF infants (Table 2). The NR3C1 (Nuclear Receptor Subfamily 3 Group C Member 1) gene encodes the glucocorticoid receptor to which cortisol and other glucocorticoids bind. The unbound receptor resides in the cytosol of the cell. When glucocorticoids bind, the NR3C1-glucocorticoid complex can either up-regulate the expression of anti-inflammatory proteins in the nucleus or repress the expression of pro-inflammatory proteins in the cytosol by preventing the translocation of other transcription factors from the cytosol into the nucleus [50]. Interestingly, NR3C1 expression was ~4.6-fold higher in BF than FF infants. Human milk contains cortisol, whereas infant formula does not. It has long been known that either systemic [51] or enterally-administered glucocorticoids [52] stimulate intestinal maturation in rodents. In addition to acting within the gut, milk glucocorticoids are absorbed into the circulation of the suckling neonate. For example, corticosterone was detectable in the serum of adrenalectomized pups fed with their own mother’s milk [51]. In humans, salivary cortisol was higher in breastfed than formula-fed infants [53] and salivary cortisol concentrations were positively correlated in breastfeeding mothers and their breastfed infants [54]. In terms of intestinal immunity, cortisone acetate decreased the immune response to both endogenous and exogenous inflammatory stimuli, in human infant intestinal xenografts implanted into mice [55]. More recently, the potential importance of milk-borne cortisol as a broader programmer of infant development has been proposed in terms of the gut-brain-axis and behavioral outcomes [56,57,58]. Given the strong association with microbial gene expression, future studies investigating mechanisms whereby milk cortisol and the microbiome interact to regulate NR3C1 signaling in infant outcomes are warranted. 

Another host immune related gene that was associated with microbial virulence gene expression was LTBP4, or latent transforming growth factor beta binding protein 4. The protein encoded by this gene binds TGF-β as it is secreted and targeted to the extracellular matrix. This protein controls TGF-β activation by binding to the latency-associated peptide, which is located in the regulatory chain of the growth factor and regulates integrin-dependent activation of TGF-β [59]. Little is known regarding its specific function in the intestine, however, recessive mutations of the LTBP4 gene caused malformations, including diverticulosis, enlargement, tortuosity, and stenosis at various levels of the intestinal tract [60]. Human observational and preclinical intervention studies have shown that TGF-β is important in developing and maintaining appropriate immune responses in the offspring. A recent review of the literature demonstrated that TGF-β delivered orally to neonatal animals showed a positive association with TGF-β1 or TGF-β2, demonstrating protection against immunologically related outcomes in 92% of the studies reviewed [61]. Similarly, a systematic review of human studies showed a positive association between TGF-β1 or TGF-β2 and protection against allergy in infants and young children [62]. Recent studies have linked commensal bacteria (e.g., Clostridiales) with supporting a TGF-β-rich environment that promotes accumulation of T-regulatory cells in the gut [63]. A recent in vitro study demonstrated that butyrate was the main bacterial metabolite that upregulated TGF-β production by intestinal epithelial cells [64]. This effect was associated with the histone deacetylase (HDAC) inhibitory activity of butyrate, rather than signaling through the G-protein coupled SCFA receptors, GPR41, GPR43 or GPR109a [64]. In contrast, specificity protein 1 (SP1) was the transcription factor that mediated the HDAC effect of butyrate on TGF-β1 production [64]. Although SP1 was not detected in the sCCA, it is a well-known regulator of gene expression throughout the digestive tract [65]. Thus, TGF-β is an important cytokine regulating neonatal immune development. It can be derived either from maternal milk or via butyrate-induced production by intestinal epithelial cells, linking the microbiota to host gene expression. We speculate that LTBP4 expression may be upregulated in the intestine of the breastfed infant as a mechanism to regulate the activity of TGF-β in the developing intestine.

Catenin delta 1 is encoded by the gene CTNND1, which was one of the barrier related genes associated with the SEEDLevel2 microbial genes. This protein, also known as p120, is a major component of multiprotein cell-cell adhesion complexes containing other catenins and epithelial cadherin (E-cadherin) [66]. It is also a tyrosine kinase substrate that has been linked to receptor signaling through the epidermal growth factor receptor, among others [67]. In a human colon adenocarcinoma cell line (HCA7), loss of p120 reduced transepithelial resistance and increased neutrophil binding and cyclooxygenase-2 activity [65]. The importance of p120 was shown in p120 conditional knock-out mice, in which p120 deficiency led to loss of cell-cell adhesion, a reduction in transepithelial resistance, and inflammation [66]. The authors concluded that p120 loss disrupts the neonatal intestinal barrier and amplifies neutrophil engagement and that these changes lead to severe inflammation during colonization of the neonatal gut [66]. Thus, up-regulation of CTNND1 in the exfoliome of BF infants supports earlier observations of enhanced maintenance of barrier function [68] and a reduction in inflammation [69] in BF infants relative to their FF counterparts. 

## 5. Conclusions

This study uses a combination of univariate and multivariate statistical approaches to identify shifts in postnatal developmental patterns in the early neonatal period. Specifically, we have demonstrated that both sCCA and sPCA can be used in support of the formulation of hypothesis-based patient-powered precision medicine studies via its ability to identify candidate genes that might be active in the host gut epithelium as well as SEEDLevel2 commensal microbiome categories that reflect the different feeding types in neonates. Our results show that these two multivariate approaches complement the testing for significant difference in host gene expression and can provide a deeper insight of the structure present in data (sPCA and sCCA) as well as identify potential interaction between the host gut epithelium and the commensal microbiota (sCCA). Furthermore, we propose that our ability to use host exfoliated cell mRNA instead of biopsy or autopsy material, in combination with microbiome-derived DNA, RNA and metabolites, will enable the development of novel predictive computational models describing host-microbe interactions associated with healthy gastrointestinal development of infants. 

## Figures and Tables

**Table 1:** Volatile fatty acid concentrations in stool of breast- and formula-fed infants. Data are presented as mean ± standard error of the mean (SEM).

Concentration (µmoles/g Dry Matter)	Breast-Fed (n = 6)	Formula-Fed (n = 6)
Short Chain Fatty Acids		
Total	233.2 ± 47.2	410.9 ± 37.5 *
Acetate	206.7 ± 51.0	327.3 ± 29.7 *
Butyrate	3.17 ± 2.33	18.7 ± 8.06 *
Propionate	13.26 ± 4.35	64.9 ± 10.9 *
Branched Chain Fatty Acids		
Total	13.35 ± 3.72	9.72± 2.42
Isobutyrate	12.70 ± 3.86	3.89 ± 0.98 *
Isovalerate	0.65 ± 0.50	4.74 ± 1.18 *
Valerate	0.0 ± 0.0	1.09 ± 0.55 *

* Indicates differences (*p* ≤ 0.05) between groups within each row.

**Table 2:** Relative expression levels of the top differentially expressed genes in three-month-old breast-fed (BF) vs. formula-fed (FF) infants.

Gene Symbol	Gene Name	Fold-Change (Mean BF/Mean FF)	*q*-Value
ARHGAP26	Rho GTPase Activating Protein 26	4.96	0.100
GPD2	Glycerol-3-Phosphate Dehydrogenase 2	4.69	0.037
NR3C1 *	Nuclear Receptor Subfamily 3, Group C, Member 1	4.65	0.039
DEFB118 *	Defensin Beta 118	3.62	0.018
PRKRA	Protein Activator of IFN Induced Protein Kinase	3.62	0.087
LTBP4 *	Latent TGF-ß Binding Protein 4	2.58	0.065
CTNND1 *	Catenin Delta 1	2.54	0.065
ARHGAP23	Rho GTPase Activating Protein 26	2.31	0.091

Abbreviations: IFN, interferon; TGF-ß, Transforming Growth Factor Beta. * indicates genes participating in one of the components identified by either sPCA or sCCA analyses.

## Supplementary Materials

The following are available online at https://www.mdpi.com/2076-2607/8/12/2032/s1, Figure S1: Data processing workflow. Figure S2: sPCA results for the microbial communities at SEEDLevel2. Figure S3: Sparse CCA of SCFA receptor-related host genes and the microbial communities at SEEDLevel2. Table S1: Microbial SEEDLevel2 categories utilized for sCCA and sPCA. Table S2: Host short chain fatty acid (SCFA) receptor-related genes utilized for sPCA and sCCA. Table S3: Host immunity and defense genes utilized for sPCA and sCCA. Table S4: Host intestinal barrier genes utilized for sCCA and sPCA. Table S5: List of host SCFA receptor-related genes that form the horizontal (first component) and the vertical (second component) axes in the sPCA plot shown in Figure 2. Table S6: List of host immunity and defense genes that form the horizontal (first component) and the vertical (second component) axes in the sPCA analysis depicted in Figure 3. Table S7a: List of host immunology and defense-related genes that form the horizontal (first component) and the vertical (second component) axes in the sCCA plot shown in Figure 4a. Table S7b: List of SEEDLevel2 microbial categories that form the horizontal (first component) and the vertical (second component) axes depicted in Figure 4b. Table S8: List of host barrier function-related genes host genes that form the horizontal (first component) and the vertical (second component) axes in the sPCA plot shown in Figure 5. Table S9a: List of barrier function-related host genes that form the horizontal (first component) and the vertical (second component) axes of the sCCA plot depicted in Figure 6a. Table S9b: List of SEEDLevel2 microbial categories that form the horizontal (first component) and the vertical (second component) axes depicted in Figure 6b. Table S10: Simulation comparison between the performance of sub-dimensional CCA, sCCA and sPCA.

## Appendix A

### Appendix A.1. The General Settings of PCA, CCA, sPCA and sCCA

PCA and sPCA: If X is an n×p data matrix of n observations from random vectors $x={\left(x_{1},x_{2},\ldots x_{p}\right)}^{T}$ containing p measured experimental variables then the loading vector $u_{1}\in {\mathbb{R}}^{p}$ of the first PC is found by maximizing the directed data variance and is the solution of

$$u_{1}=\underset{u}{\mathrm{argmax}}\;u^{T}\left(X^{T}X\right)u,\;\mathrm{subject}\;\mathrm{to}\;u^{T}u\leq 1$$

The loading vectors of the subsequent PCs can be found sequentially by removing the variance of the preceding ones. When the dimension of data p is larger than the sample size n there is little hope of performing inferential interpretation with very limited data from the standard PCA. In those cases, the sparse version of PCA, sPCA can be used to extract the variation information resulting in a set of sparse PC vectors that explain a maximum amount of variance. The PCs are found as a solution to the above optimization problem with an additional sparse constraint $∥u{∥}_{1}\leq c$, where $∥\cdot {∥}_{1}$ is $l_{1}$–norm and the tuning parameter *c* is positive [17,28].

CCA and sCCA: The general setting for classical CCA is as follows: If X and Y are *n* × *p* and *n* × *q* data matrices recording *n* observations from random vectors *x* = (x_1_, x_2_,…, x_p_)^T^ and *y* = (y_1_, y_2_,…, y_q_)^T^, respectively then CCA aims to find projection directions *u*_1_ ∈ ℝ^p^ and *v*_1_ ∈ ℝ^q^ such that

$$\left(u_{1},v_{1}\right)=arg\;\underset{u,v}{\max}Corr\left(u^{T}x,v^{T}y\right)=arg\;\underset{u,v}{\max}\frac{u^{T}\sum_{xy}^{\;}v}{\sqrt{(u^{T}\sum_{xx}u)\left(v^{T}\sum_{yy}v\right)}}$$

where Σ_xy_, Σ_xx_, and Σ_yy_ are covariance and variance matrices. The empirical version of above objective function can be written as:

$$\underset{u,v}{\max}u^{T}X^{T}Yv$$

subject to the constraints:

$$u^{T}\frac{X^{T}X}{n}u\leq 1,\;v^{T}\frac{Y^{T}Y}{n}v\;\leq 1$$

However, this objective function does not lead to a solution in a closed form when the sample size ***n*** is less than min (*p; q*). Thus, sCCA added regularization conditions allow for a sparse solution to the above optimization problem.

$$∥u{∥}_{1}\leq c_{1},\;∥v{∥}_{1}\leq c_{2},$$

Where ‖.‖_1_ denotes the *l*_1_ –norm and the tuning parameters *c*_1_ and *c*_2_ are positive and control the sparsity of the solution. These parameters were chosen based on findings of a permutation testing procedure [17]. The algorithm to solve this constrained optimization problem is related to soft-thresholding and binary search procedures [17,70].

### Appendix A.2. Synthetically Generated Data Analysis to Compare the Performance of sCCA, sPCA, and Sub-Dimensional CCA

To compare the performance of sCCA vs sPCA and the sub-dimensional CCA [14], we utilized an approach based on a latent variables model from [30] for our simulation study (Table S10). The results of these simulations were used to inform our selection of methods for integrative data analysis. Specifically, we generated data matrices X and Y where the dependency between these two sets of variables was induced by a latent random variable ζ and the covariances in *x*, *y* can be explained in part by ζ. We assumed *x* = ζ*w*_x_ + ε_x_ and *y* = ζ*w*_y_ + ε_y_, where ζ ∈ N(0; σ_ζ_^2^), and ε_x_, ε_y_ were random noise vectors that followed N(0; σ_ε_^2^I) and that there were p and q total number of features, i.e., the dimensionality of the vectors *x* and *y* respectively. Furthermore, *w*_x_ ∈ ℝ^p^ and *w*_y_ ∈ ℝ^q^ were column vectors of preset weights where without loss of generality their entry-wise sum was assumed to be 1. We also assumed that the first p_0_ and q_0_ elements of *w*_x_ and *w*_y_ were non-zero and interpreted them as the relevant features or ground truth in the simulations. For each simulated data set, we used *n* to represent the sample size. During the evaluation, we used a permutation test [17] to select the sCCA tuning parameters and subsequently summarized the output by the true positive rate (TPR) and false positive rate (FPR) to measure the identification performance for the relevant entries in *x* and *y*.

$$TPR=\;\frac{TP}{TP+FN};FPR=\;\frac{FP}{FP+TN}$$

where TP, FP, TN, and FN are the numbers of true positives, false positives, true negatives, and false negatives related to the ground truth features in the simulated data. In our simulations, we considered only the case p_0_ = q_0_ = 10 where p_0_, and q_0_ represent the true number of host genes/features that are associated with the microbiota, and the true number of microbial species/features that are associated with the host transcriptome, respectively. Similar to [71], in each simulation, the non-zero values in *w*_x_ and *w*_y_ were set to be equal to 1/p_0_ and 1/q_0_, respectively. In addition, we set σ_ε_ = 1, σ_ζ_ = 6, and allowed p, q, and n to vary in each simulation study to generate 100 times the “observed” data sets *X* and *Y*. A summary of the mean TPR, the TPR standard error, and FPR on sub-dimensional CCA, sCCA, and sPCA is provided in Table S10. Note that, when p is large, the sub-dimensional CCA is infeasible because of its high computational complexity. These findings confirm our expectation that integrative analysis provided by the sCCA has the potential to outperform both sub-dimensional CCA and sPCA. This is especially important in situations where the prior knowledge suggests that the two data sets are derived from interrelated processes, such as the interplay between diet, gut microbiota, and host transcriptome [72,73,74].
