# Supplementary material for: Assessing the Multivariate Relationship between the Human Infant Intestinal Exfoliated Cell Transcriptome (Exfoliome) and Microbiome in Response to Diet
Source: Microorganisms. 2020 Dec 18;8(12):2032. doi: 10.3390/microorganisms8122032 (PMC7766018; doi:10.3390/microorganisms8122032)
Supplement: Supplementary file 1 [file microorganisms-08-02032-s001.zip › Suppl material final/Supplemental Figures R1.pdf]

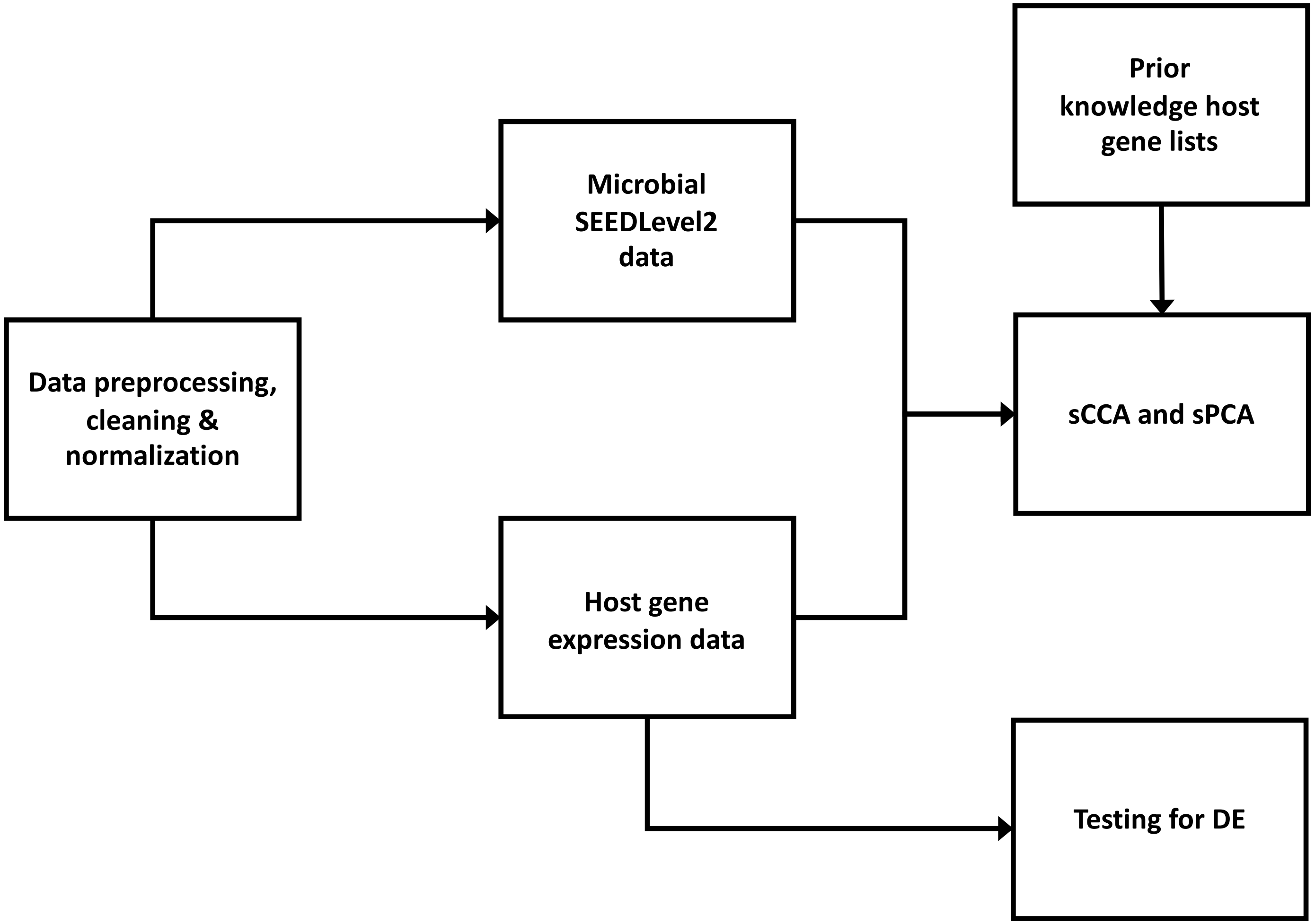

Figure S1. Data processing work flow.

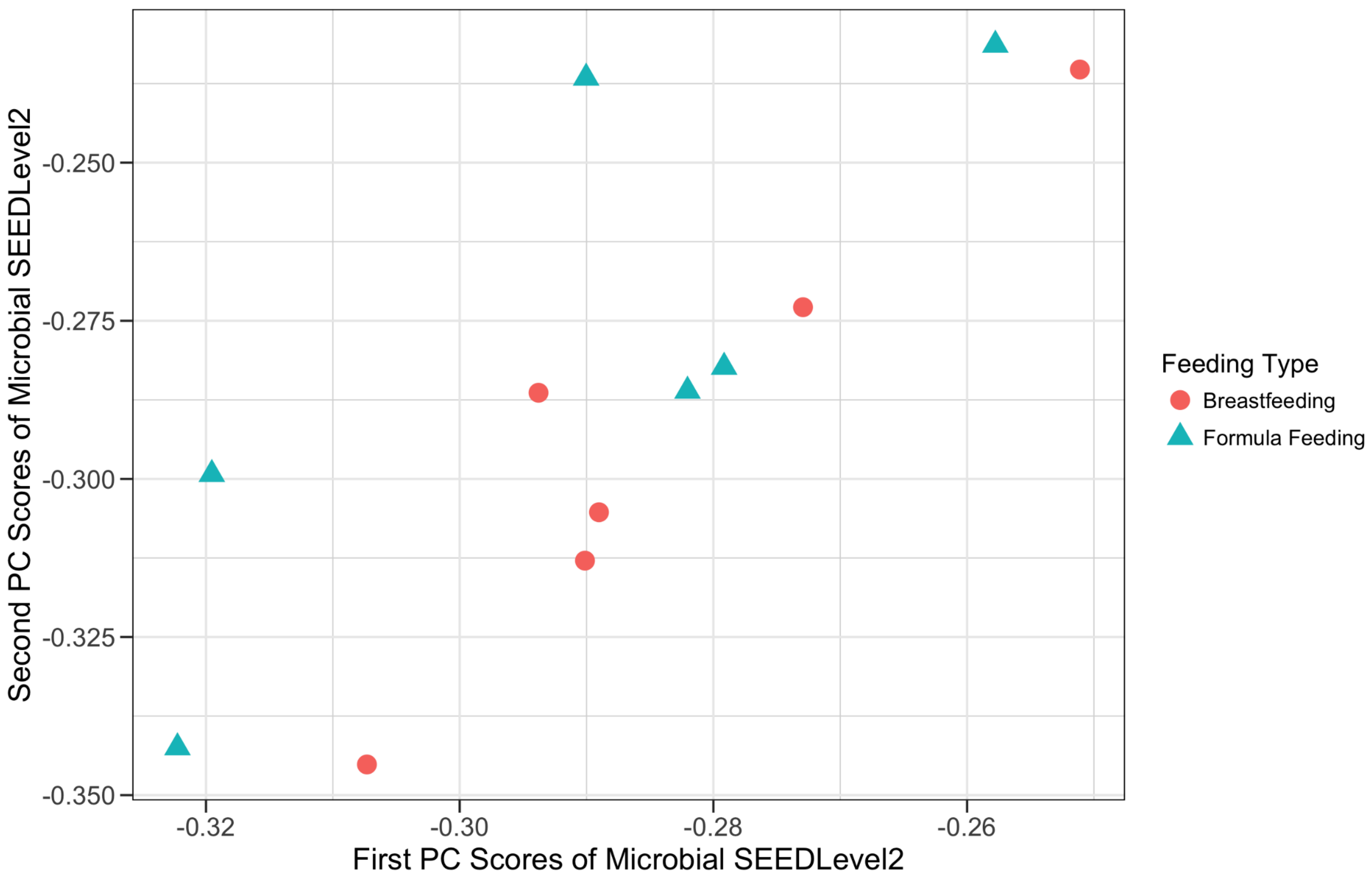

Figure S2. sPCA results for the microbial communities at SEEDLevel2. The x-axis and the y-axis represent the first and the second principal components, respectively, obtained after the sPCA application to the normalized data from the SEEDLevel2 subsystem of microbial communities.

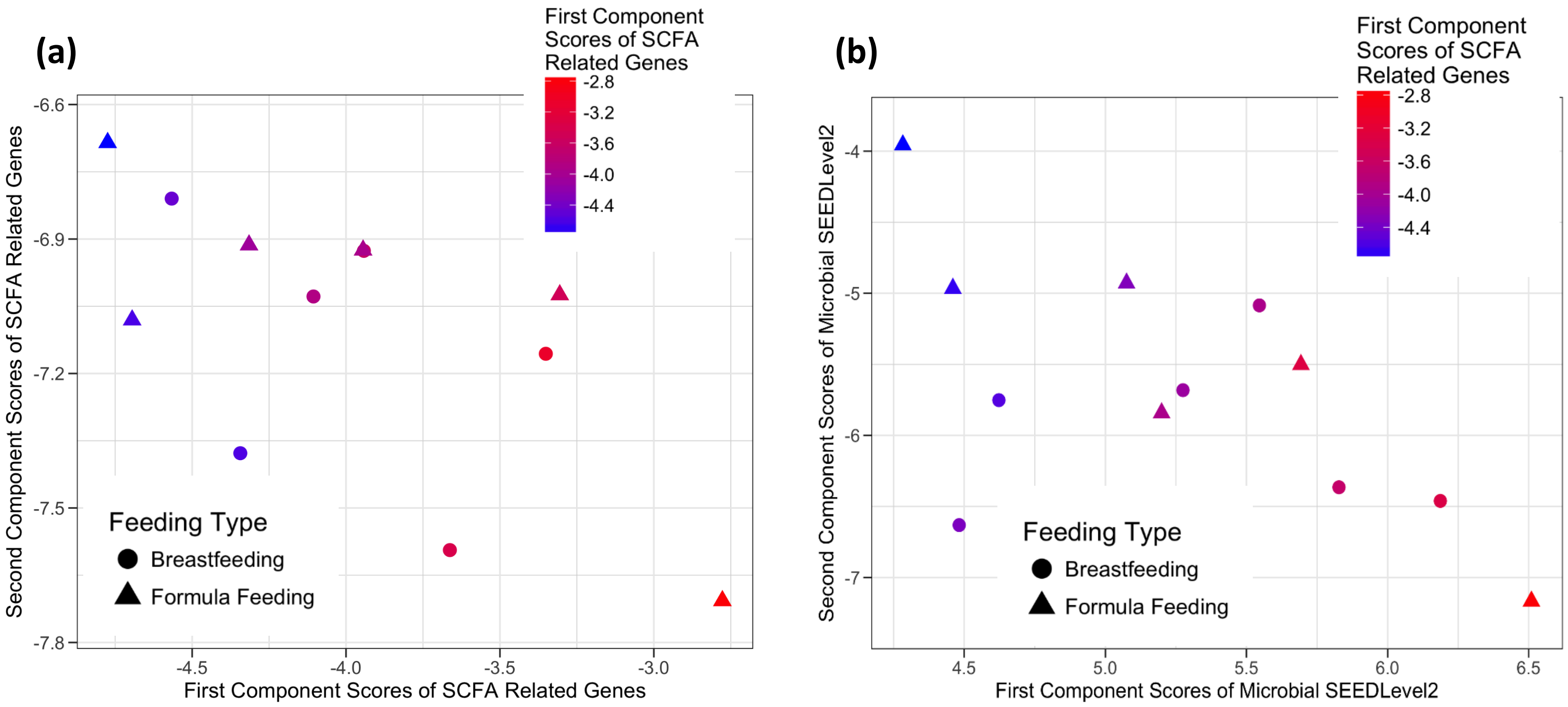

Figure S3. Sparse CCA of SCFA receptor-related host genes and the microbial communities at SEEDLevel2. In panel (**a**), the x-axis and the y-axis represent the first and the second component scores, respectively, from the normalized microarray expression of the SCFA related host genes. Coloring of the sample points represents the first component scores from the microbial communities from the SEEDLevel2 subsystem. In panel (**b**), the x-axis and the y-axis represent the first and the second component scores from the normalized SEEDLevel2 subsystem of microbial communities, respectively. Coloring of the sample points represents the first component scores from the normalized microarray expression of the SCFA genes related host genes.
