# Supplementary material for: Assessing the Multivariate Relationship between the Human Infant Intestinal Exfoliated Cell Transcriptome (Exfoliome) and Microbiome in Response to Diet
Source: Microorganisms. 2020 Dec 18;8(12):2032. doi: 10.3390/microorganisms8122032 (PMC7766018; doi:10.3390/microorganisms8122032)
Supplement: Supplementary file 1 [file microorganisms-08-02032-s001.zip › Suppl material final/Supplemental Tables .docx]

**Supplementary Materials**

**Table S1.** Microbial SEEDLevel2 categories utilized for sCCA and sPCA.

| **SEEDLevel2 Categories** |
| --- |
| Amino Acids and Derivatives_Alanine, serine, and glycine |
| Amino Acids and Derivatives_Arginine; urea cycle, polyamines |
| Amino Acids and Derivatives_Aromatic amino acids and derivatives |
| Amino Acids and Derivatives_Branched-chain amino acids |
| Amino Acids and Derivatives_Glutamine, glutamate, aspartate, asparagine; ammonia assimilation |
| Amino Acids and Derivatives_Histidine Metabolism |
| Amino Acids and Derivatives_Lysine, threonine, methionine, and cysteine |
| Amino Acids and Derivatives_Osmotic stress |
| Amino Acids and Derivatives_Proline and 4-hydroxyproline |
| Carbohydrates_Aminosugars |
| Carbohydrates_Carbohydrates |
| Carbohydrates_Central carbohydrate metabolism |
| Carbohydrates_Clustering-based subsystems |
| Carbohydrates_CO2 fixation |
| Carbohydrates_Di- and oligosaccharides |
| Carbohydrates_Fermentation |
| Carbohydrates_Monosaccharides |
| Carbohydrates_One-carbon Metabolism |
| Carbohydrates_Organic acids |
| Carbohydrates_Sugar alcohols |
| Carbohydrates_Uptake system |
| Cell Division and Cell Cycle_Cell cycle in Prokaryota |
| Cell Division and Cell Cycle_Cell Division and Cell Cycle |
| Cell Wall and Capsule_Capsular and extracellular polysacchrides |
| Cell Wall and Capsule_Cell Wall and Capsule |
| Cell Wall and Capsule_Gram-Negative cell wall components |
| Cell Wall and Capsule_Gram-Positive cell wall components |
| Clustering-based subsystems_Biosynthesis of galactoglycans and related lipopolysacharides |
| Clustering-based subsystems_Cell Division |
| Clustering-based subsystems_Cluster of Unknown Function |
| Clustering-based subsystems_Clustering-based subsystems |
| Clustering-based subsystems_Cytochrome biogenesis |
| Clustering-based subsystems_D-tyrosyl-tRNA(Tyr) deacylase (EC 3.1.-.-) cluster |
| Clustering-based subsystems_DNA polymerase III epsilon cluster |
| Clustering-based subsystems_Fatty acid metabolic cluster |
| Clustering-based subsystems_Hypothetical in Lysine biosynthetic cluster |
| Clustering-based subsystems_Hypothetical lipase related to Phosphatidate metabolism |
| Clustering-based subsystems_Hypothetical protein possible functionally linked with Alanyl-tRNA synthetase |
| Clustering-based subsystems_Isoprenoid/cell wall biosynthesis: PREDICTED UNDECAPRENYL DIPHOSPHATE PHOSPHATASE |
| Clustering-based subsystems_Lipoprotein B cluster |
| Clustering-based subsystems_Lysine, threonine, methionine, and cysteine |
| Clustering-based subsystems_Probably GTP or GMP signaling related |
| Clustering-based subsystems_Probably organic hydroperoxide resistance related hypothetical protein |
| Clustering-based subsystems_Probably Pyrimidine biosynthesis-related |
| Clustering-based subsystems_Probably Ybbk-related hypothetical membrane proteins |
| Clustering-based subsystems_Protein export? |
| Clustering-based subsystems_Putative asociate of RNA polymerase sigma-54 factor rpoN |
| Clustering-based subsystems_Putrescine/GABA utilization cluster-temporal,to add to SSs |
| Clustering-based subsystems_Recombination related cluster |
| Clustering-based subsystems_recX and regulatory cluster |
| Clustering-based subsystems_Related to N-acetylglucosamine utilization subsystem? |
| Clustering-based subsystems_Ribosome-related cluster |
| Clustering-based subsystems_TldD cluster |
| Clustering-based subsystems_Translation |
| Clustering-based subsystems_Tricarboxylate transporter |
| Clustering-based subsystems_tRNA sulfuration |
| Cofactors, Vitamins, Prosthetic Groups, Pigments_Biotin |
| Cofactors, Vitamins, Prosthetic Groups, Pigments_Coenzyme A |
| Cofactors, Vitamins, Prosthetic Groups, Pigments_Fe-S clusters |
| Cofactors, Vitamins, Prosthetic Groups, Pigments_Folate and pterines |
| Cofactors, Vitamins, Prosthetic Groups, Pigments_Isoprenoids |
| Cofactors, Vitamins, Prosthetic Groups, Pigments_Lipoic acid |
| Cofactors, Vitamins, Prosthetic Groups, Pigments_NAD and NADP |
| Cofactors, Vitamins, Prosthetic Groups, Pigments_Pyridoxine |
| Cofactors, Vitamins, Prosthetic Groups, Pigments_Quinone cofactors |
| Cofactors, Vitamins, Prosthetic Groups, Pigments_Riboflavin, FMN, FAD |
| Cofactors, Vitamins, Prosthetic Groups, Pigments_Tetrapyrroles |
| Cofactors, Vitamins, Prosthetic Groups, Pigments_Thiamine and thiamine pyrophosphate |
| DNA Metabolism_DNA Metabolism |
| DNA Metabolism_DNA repair |
| DNA Metabolism_DNA replication |
| Fatty Acids and Lipids_Fatty acids |
| Fatty Acids and Lipids_Fatty Acids and Lipids |
| Fatty Acids and Lipids_Phospholipids |
| Fatty Acids and Lipids_Triacylglycerols |
| Membrane Transport_ABC transporters |
| Membrane Transport_Membrane Transport |
| Membrane Transport_Uni- Sym- and Antiporters |
| Miscellaneous_Miscellaneous |
| Motility and Chemotaxis_Flagellar motility in Prokaryota |
| Motility and Chemotaxis_Motility and Chemotaxis |
| Nitrogen Metabolism_Nitrogen Metabolism |
| Nucleosides and Nucleotides_Detoxification |
| Nucleosides and Nucleotides_Nucleosides and Nucleotides |
| Nucleosides and Nucleotides_Purines |
| Nucleosides and Nucleotides_Pyrimidines |
| Phosphorus Metabolism_Phosphorus Metabolism |
| Potassium metabolism_Potassium metabolism |
| Protein Metabolism_Lipoproteins |
| Protein Metabolism_Protein biosynthesis |
| Protein Metabolism_Protein degradation |
| Protein Metabolism_Protein folding |
| Protein Metabolism_Protein processing and modification |
| Protein Metabolism_Secretion |
| Protein Metabolism_Selenoproteins |
| Regulation and Cell signaling_Regulation and Cell signaling |
| Respiration_ATP synthases |
| Respiration_Electron accepting reactions |
| Respiration_Electron donating reactions |
| Respiration_Respiration |
| RNA Metabolism_RNA processing and modification |
| RNA Metabolism_Transcription |
| Secondary Metabolism_Aromatic amino acids and derivatives |
| Stress Response_Detoxification |
| Stress Response_Osmotic stress |
| Stress Response_Oxidative stress |
| Stress Response_Periplasmic Stress |
| Stress Response_Stress Response |
| Sulfur Metabolism_Organic sulfur assimilation |
| Sulfur Metabolism_Sulfur Metabolism |
| Unclassified_Unclassified |
| Virulence_Iron Scavenging Mechanisms |
| Virulence_Resistance to antibiotics and toxic compounds |
| Virulence_Type III, Type IV, ESAT secretion systems |
| Virulence_Virulence |

**Table S2.** Host short chain fatty acid (SCFA) receptor-related genes utilized for sPCA and sCCA.

| **Gene ID** | |
| --- | --- |
| *BAK1* | *HCAR2* |
| *BAX* | *HSPA12A* |
| *BCL2L1* | *HSPA12B* |
| *BLK* | *HSPA14* |
| *BRD2* | *HSPA1A* |
| *CAMK1D* | *HSPA1B* |
| *CAMK2B* | *HSPD1* |
| *CASK* | *ICAM1* |
| *CASP4* | *ICK* |
| *CCNA1* | *KLF4* |
| *CCNB1* | *LCK* |
| *CCND1* | *MAP2K2* |
| *CDK2* | *MCT-1* |
| *CDK4* | *MCT-4* |
| *CDK7* | *MFGE8* |
| *CDK8* | *MOATS* |
| *CDKN1A* | *MTCO3* |
| *CHEK1* | *MUC2* |
| *CNR1* | *MUC5AC* |
| *CNR2* | *NOS2* |
| *DDR2* | *NOS2A* |
| *EGF* | *NOS3* |
| *ERBB2* | *PFTK2* |
| *FFA2* | *PLAU* |
| *FFA4* | *PRKCA* |
| *FFAR2* | *PTEN* |
| *FFAR3* | *PTPN6* |
| *FOS* | *RIPK1* |
| *FOXO3* | *ROCK1* |
| *FRAP1* | *SLC5A12* |
| *GAP43* | *SLC5A8* |
| *GPR109A* | *SMCT1* |
| *GPR109B* | *SYN1* |
| *GPR41* | *TCF4* |
| *GPR43* | *TNK2* |
| *GSK3A* | *TYRO3* |

**Table S3.** Host immunity and defense genes utilized for sCCA and sPCA.

| **Gene ID** | | | | | | |
| --- | --- | --- | --- | --- | --- | --- |
| *A1BG* | *ARHGAP20* | *C3* | *CD1C* | *CLEC2B* | *CTSK* | *DEFB129* |
| *ABCB10* | *ARHGAP21* | *C3AR1* | *CD1E* | *CLEC4E* | *CTSL2* | *DLC1* |
| *ABCC10* | *ARHGAP22* | *C7* | *CD200* | *CLEC4F* | *CTSS* | *DNAJA1* |
| *ABCC11* | *ARHGAP23* | *C8B* | *CD207* | *CLEC7A* | *CX3CL1* | *DNAJA3* |
| *ABCC3* | *ARHGAP25* | *CACYBP* | *CD22* | *CMA1* | *CX3CR1* | *DNAJA4* |
| *ABCC5* | *ARHGAP26* | *CAMLG* | *CD244* | *CNIH2* | *CXCL1* | *DPH2* |
| *ABCC6* | *ARHGAP28* | *CARD8* | *CD28* | *CNIH3* | *CXCL10* | *DUOX2* |
| *ABCC8* | *ARHGAP29* | *CARD9* | *CD34* | *COL14A1* | *CXCL11* | *DUSP16* |
| *ABHD14B* | *ARHGAP30* | *CASP1* | *CD3D* | *COL1A1* | *CXCL12* | *E2F5* |
| *ACTA2* | *ARHGAP4* | *CASP8* | *CD3E* | *COL1A2* | *CXCL13* | *EDA* |
| *ACVR1* | *ARHGAP5* | *CCL1* | *CD3G* | *COL3A1* | *CXCL16* | *EDG1* |
| *ACVR1B* | *ARHGAP6* | *CCL14* | *CD40* | *COL4A1* | *CXCL2* | *EGF* |
| *ACVR2A* | *ARHGAP9* | *CCL16* | *CD40LG* | *COL4A3* | *CXCL3* | *EGFR* |
| *ACVR2B* | *ASGR2* | *CCL17* | *CD47* | *COL5A1* | *CXCL5* | *EIF2AK2* |
| *ADORA2A* | *ATG16L1* | *CCL18* | *CD48* | *COL5A2* | *CXCL6* | *EIF2AK3* |
| *ADORA3* | *ATM* | *CCL20* | *CD59* | *COL5A3* | *CXCL9* | *ELK1* |
| *ADRB3* | *AXL* | *CCL21* | *CD6* | *COLEC10* | *CXCR3* | *ELK4* |
| *AGER* | *B2M* | *CCL22* | *CD63* | *COLEC11* | *CXCR4* | *EMR2* |
| *AHSG* | *BCL6* | *CCL23* | *CD7* | *COLEC12* | *CXCR6* | *ENG* |
| *AIM2* | *BCL9* | *CCL24* | *CD74* | *COQ10A* | *CYBB* | *EP300* |
| *AIRE* | *BCL9L* | *CCL25* | *CD79A* | *COQ10B* | *CYSLTR2* | *EPHX1* |
| *ALOX12* | *BDKRB2* | *CCL26* | *CD80* | *CPN2* | *DAPP1* | *F12* |
| *ALOX5* | *BLK* | *CCL3* | *CD81* | *CR2* | *DDT* | *F13A1* |
| *AMHR2* | *BMPR1A* | *CCL4* | *CD82* | *CRB1* | *DDX58* | *F13B* |
| *ANGPT1* | *BMPR1B* | *CCL5* | *CD86* | *CRB2* | *DEFA4* | *F3* |
| *ANTXR1* | *BMPR2* | *CCL7* | *CD8A* | *CRB3* | *DEFA5* | *F9* |
| *ANTXR2* | *BPHL* | *CCL8* | *CD9* | *CREBBP* | *DEFB1* | *FADD* |
| *ANXA8* | *BPI* | *CCNL2* | *CD96* | *CRNN* | *DEFB103A* | *FASLG* |
| *AOC2* | *BPIL1* | *CCR2* | *CD97* | *CRP* | *DEFB104A* | *FBXO42* |
| *AOC3* | *BTK* | *CCR4* | *CDH1* | *CSF1* | *DEFB105A* | *FCAR* |
| *APLN* | *C1QA* | *CCR5* | *CDX2* | *CSF2* | *DEFB106A* | *FCER2* |
| *APOH* | *C1QL1* | *CCR7* | *CEACAM6* | *CSF3* | *DEFB108B* | *FCGR2A* |
| *ARHGAP1* | *C1QL2* | *CCR8* | *CEBPB* | *CSNK2A1* | *DEFB118* | *FCGRT* |
| *ARHGAP10* | *C1QL4* | *CCR9* | *CES2* | *CSNK2A2* | *DEFB119* | *FCRL1* |
| *ARHGAP15* | *C1QTNF1* | *CCRL2* | *CHUK* | *CTGF* | *DEFB123* | *FCRL3* |
| *ARHGAP17* | *C1QTNF6* | *CD14* | *CIITA* | *CTHRC1* | *DEFB124* | *FGA* |
| *ARHGAP18* | *C1RL* | *CD180* | *CISH* | *CTNNB1* | *DEFB125* | *FGB* |
| *ARHGAP19* | *C1S* | *CD1B* | *CLCF1* | *CTSG* | *DEFB127* | *FGF10* |
| *FGF2* | *GPX1* | *HSFY1* | *IL10* | *IL27* | *JAK2* | *LTB* |
| *FGF7* | *GPX3* | *HSH2D* | *IL10RA* | *IL27RA* | *JUN* | *LTB4R* |
| *FGFR1* | *GRB2* | *HSPA1A* | *IL10RB* | *IL28RA* | *KIR3DL2* | *LTB4R2* |
| *FGFR1OP2* | *GSG2* | *HSPA1B* | *IL12A* | *IL2RA* | *KIT* | *LTBP1* |
| *FGFR2* | *GSTA2* | *HSPA1L* | *IL12B* | *IL2RB* | *KLF6* | *LTBP2* |
| *FGFR3* | *GSTM1* | *HSPA6* | *IL12RB1* | *IL4* | *KLRF1* | *LTBP4* |
| *FGG* | *GSTM3* | *HSPA8* | *IL12RB2* | *IL4R* | *KNG1* | *LTBR* |
| *FKBP3* | *GSTM4* | *HSPB1* | *IL13* | *IL5* | *LAIR2* | *LY96* |
| *FKBP4* | *GSTM5* | *HSPB3* | *IL13RA1* | *IL5RA* | *LCN2* | *LYN* |
| *FKBP5* | *GSTO1* | *HSPB7* | *IL15* | *IL6* | *LEF1* | *LYZL6* |
| *FLT3LG* | *GSTP1* | *HSPB8* | *IL17B* | *IL6R* | *LEMD1* | *MAEA* |
| *FOS* | *GSTT2* | *HSPD1* | *IL17C* | *IL6ST* | *LGALS2* | *MAL* |
| *FOSL1* | *GULP1* | *HYOU1* | *IL17D* | *IL7R* | *LGALS3* | *MALT1* |
| *FOXP3* | *GYPC* | *ICAM1* | *IL17F* | *IL8* | *LGALS3BP* | *MAP2K3* |
| *FUT3* | *GZMH* | *ICAM2* | *IL17RB* | *IL8RA* | *LGALS4* | *MAP2K4* |
| *FUT6* | *HBEGF* | *ICAM3* | *IL17RC* | *IL8RB* | *LGALS8* | *MAP3K1* |
| *FUT7* | *HCK* | *ICAM4* | *IL17RD* | *IL9R* | *LIF* | *MAP3K10* |
| *FYN* | *HDAC4* | *ICEBERG* | *IL17RE* | *IRAK2* | *LIFR* | *MAP3K11* |
| *FZD1* | *HFE* | *ICOS* | *IL18* | *IRF1* | *LITAF* | *MAP3K7IP1* |
| *FZD3* | *HGF* | *IER3* | *IL18RAP* | *IRF2* | *LMO1* | *MAP3K9* |
| *FZD4* | *HLA-DMA* | *IFI30* | *IL19* | *IRF3* | *LMO2* | *MAP4K4* |
| *FZD6* | *HLA-DMB* | *IFI35* | *IL1A* | *IRF5* | *LPO* | *MAPK1* |
| *FZD7* | *HLA-DOB* | *IFIH1* | *IL1B* | *IRF7* | *LRCH1* | *MAPK3* |
| *FZD8* | *HLA-DPA1* | *IFIT2* | *IL1F10* | *IRF8* | *LRCH3* | *MAPK8* |
| *FZD9* | *HLA-DQA1* | *IFIT5* | *IL1F5* | *ISG20* | *LRCH4* | *MAPK8IP3* |
| *GAB1* | *HLA-DQB2* | *IFITM1* | *IL1F6* | *ITGA2* | *LRMP* | *MAPKAPK2* |
| *GAB2* | *HLA-DRA* | *IFITM2* | *IL1F7* | *ITGA3* | *LRP5* | *MCFD2* |
| *GAB3* | *HLA-DRB5* | *IFITM3* | *IL1F8* | *ITGA4* | *LRP6* | *MERTK* |
| *GADD45A* | *HLA-E* | *IFNA1* | *IL1F9* | *ITGA5* | *LRRC15* | *MET* |
| *GADD45B* | *HLA-F* | *IFNA17* | *IL1R1* | *ITGA6* | *LRRC28* | *MICA* |
| *GATA3* | *HLA-G* | *IFNA2* | *IL1R2* | *ITGAV* | *LRRC40* | *MMD* |
| *GBP1* | *HMGB1* | *IFNAR2* | *IL1RL2* | *ITGB1* | *LRRC57* | *MMP1* |
| *GBP2* | *HRAS* | *IFNB1* | *IL1RN* | *ITGB2* | *LRRC59* | *MMP13* |
| *GIMAP5* | *HRH1* | *IFNGR1* | *IL20* | *ITGB3* | *LRRC7* | *MMP3* |
| *GIMAP6* | *HRH4* | *IGBP1* | *IL21R* | *ITGB5* | *LRRC8A* | *MMP7* |
| *GIMAP7* | *HS3ST1* | *IGF1* | *IL22* | *ITGB6* | *LRRC8D* | *MMP9* |
| *GIMAP8* | *HS3ST2* | *IGLL1* | *IL22RA2* | *ITPR1* | *LRRC8E* | *MOCOS* |
| *GLI1* | *HS3ST3A1* | *IKBKAP* | *IL23A* | *ITPR2* | *LRRK2* | *MOSC1* |
| *GLI2* | *HS3ST3B1* | *IKBKB* | *IL23R* | *ITPR3* | *LRSAM1* | *MPST* |
| *GLI3* | *HSF1* | *IKBKE* | *IL24* | *JAK1* | *LTA* | *MPV17* |
| *MR1* | *OAS2* | *PRG2* | *S100A9* | *SNED1* | *TGFB2* | *TSPAN4* |
| *MRVI1* | *OASL* | *PRG3* | *SAA2* | *SOCS1* | *TGFB3* | *TST* |
| *MSRA* | *OSCAR* | *PRKAB1* | *SAA4* | *SOCS3* | *TGFBR1* | *TYRO3* |
| *MSRB3* | *OTUB2* | *PRKAG2* | *SARM1* | *SOD1* | *TGFBR2* | *TYROBP* |
| *MUC1* | *PAFAH2* | *PRKAG3* | *SCARB1* | *SOD2* | *TGFBR3* | *UBE2N* |
| *MYD88* | *PARK7* | *PRKRA* | *SCYE1* | *SP1* | *TGFBRAP1* | *UBE2V1* |
| *NCR1* | *PDGFA* | *PROC* | *SDC4* | *SP2* | *THBS1* | *ULBP1* |
| *NCSTN* | *PDGFRA* | *PROCR* | *SELENBP1* | *SP3* | *THBS2* | *ULBP2* |
| *NDST1* | *PDGFRB* | *PROZ* | *SEMA4A* | *SP4* | *THBS3* | *ULBP3* |
| *NFAT5* | *PELI1* | *PSEN1* | *SEMA4D* | *SPACA3* | *THBS4* | *VANGL2* |
| *NFATC1* | *PF4* | *PSEN2* | *SEMA5A* | *SPI1* | *TICAM1* | *VAV2* |
| *NFATC2* | *PGLYRP1* | *PSENEN* | *SEMA5B* | *SQSTM1* | *TICAM2* | *VAV3* |
| *NFATC3* | *PGLYRP4* | *PTAFR* | *SEPP1* | *SRC* | *TIMP1* | *VTCN1* |
| *NFATC4* | *PILRB* | *PTCHD2* | *SEPX1* | *STAT2* | *TIRAP* | *VTN* |
| *NFKB1* | *PLA2G4F* | *PTEN* | *SERHL* | *STAT3* | *TLR10* | *VWF* |
| *NFKB2* | *PLAT* | *PTGES3* | *SERP1* | *STAT4* | *TLR2* | *WAS* |
| *NFKBIA* | *PLAUR* | *PTGS1* | *SERPINE1* | *STAT5A* | *TLR3* | *WASL* |
| *NFKBIB* | *PLG* | *PTGS2* | *SH3BP4* | *STAT5B* | *TLR4* | *WNT5A* |
| *NFKBIL1* | *PLSCR1* | *PTK2* | *SIGIRR* | *STAT6* | *TLR5* | *XBP1* |
| *NFRKB* | *PLSCR4* | *PTK2B* | *SIGLEC1* | *SUFU* | *TLR7* | *XCL1* |
| *NKG7* | *PLUNC* | *PTPN22* | *SLA* | *SWAP70* | *TLR8* | *XCR1* |
| *NKTR* | *PON2* | *PTPRC* | *SLAMF6* | *SYK* | *TLR9* | *XRCC3* |
| *NMI* | *PPARA* | *PVRL2* | *SLAMF7* | *TACR1* | *TMPO* | *XRCC5* |
| *NOTCH1* | *PPARD* | *PVRL3* | *SLAMF8* | *TACR3* | *TNF* | *XRCC6* |
| *NOTCH2* | *PPARG* | *PVRL4* | *SLAMF9* | *TAPBPL* | *TNFAIP8* | *YY1* |
| *NOTCH2NL* | *PPIA* | *PYGO2* | *SLC15A1* | *TBK1* | *TNFAIP8L1* |  |
| *NOTCH3* | *PPIB* | *RAC1* | *SLC16A1* | *TBX21* | *TNFAIP8L2* |  |
| *NOTCH4* | *PPIF* | *RAET1E* | *SLC22A5* | *TBXA2R* | *TNFAIP8L3* |  |
| *NOX1* | *PPIH* | *RBL1* | *SLIT3* | *TBXAS1* | *TNFRSF1A* |  |
| *NOX5* | *PPIL1* | *RBL2* | *SLURP1* | *TCF7* | *TNFRSF1B* |  |
| *NOXA1* | *PPIL2* | *REG3G* | *SMAD1* | *TCF7L1* | *TNFRSF8* |  |
| *NPTX2* | *PPIL3* | *REL* | *SMAD2* | *TCF7L2* | *TNFRSF9* |  |
| *NPTXR* | *PPIL4* | *RELA* | *SMAD3* | *TCIRG1* | *TNFSF14* |  |
| *NR2C2* | *PPP2R2A* | *RGMA* | *SMAD4* | *TEC* | *TNKS* |  |
| *NR3C1* | *PPP2R2B* | *RGS1* | *SMAD5* | *TFCP2* | *TOLLIP* |  |
| *NR4A1* | *PPP2R2D* | *RHOA* | *SMAD6* | *TFCP2L1* | *TPSG1* |  |
| *NR4A2* | *PPP3CA* | *RIPK2* | *SMAD7* | *TFPI* | *TPT1* |  |
| *NR4A3* | *PPP3CB* | *RNPEP* | *SMAD9* | *TFPI2* | *TRAF6* |  |
| *NUP88* | *PRDX2* | *RORC* | *SMG1* | *TGFA* | *TRAP1* |  |
| *OAS1* | *PRDX6* | *S100A8* | *SMO* | *TGFB1* | *TSLP* |  |

**Table S4.** Host intestinal barrier genes utilized for sCCA and sPCA

| **Gene ID** | |
| --- | --- |
| *ACTA1* | *MUC2* |
| *ACTA2* | *MYH2* |
| *ACTB* | *MYO9B* |
| *ACTC1* | *OLfr78* |
| *ACTG1* | *PARD3* |
| *ACTG2* | *PPARG* |
| *ANGPTL4* | *PVRL1* |
| *CCL2* | *SLC9A2* |
| *CDH1* | *SLC9A3* |
| *CDH2* | *TNF* |
| *CGNL1* | *ZGLP1* |
| *CLDN1* | *ZO1* |
| *CLDN2* | *ZO2* |
| *CLDN3* | *ZO3* |
| *CLDN4* |  |
| *CTNNA1* |  |
| *CTNNA2* |  |
| *CTNNB1* |  |
| *CTNND1* |  |
| *CTNND2* |  |
| *CXCL1* |  |
| *CXCL2* |  |
| *F11R* |  |
| *FFAR1* |  |
| *FFAR2* |  |
| *FFAR3* |  |
| *FOS* |  |
| *GCG* |  |
| *GPR42* |  |
| *IFNG* |  |
| *IKBKG* |  |
| *IL17A* |  |
| *IL1B* |  |
| *IL6* |  |
| *JAM3* |  |
| *JUN* |  |
| *MAGI2* |  |
| *MLLT4* |  |

**Table S5.** Host SCFA receptor-related genes that form the horizontal (first component) and the vertical (second component) axes in the sPCA plot shown in Figure **2**.

| **Genes ID** | **Gene Name** | **Mean BF/Mean FF*** |
| --- | --- | --- |
| **First component genes** |  |  |
| *BAX* | BCL2 associated X, apoptosis regulator | 0.98 |
| *CAMK1D* | Calcium/calmodulin dependent protein kinase ID | 0.93 |
| *CAMK2B* | Calcium/calmodulin dependent protein kinase II beta | 1.10 |
| *CCNB1* | Cyclin B1 | 1.14 |
| *CCND1* | Cyclin D1 | 0.92 |
| *CDK8* | Cyclin dependent kinase 8 | 1.01 |
| *CDKN1A* | Cyclin dependent kinase inhibitor 1A | 1.30 |
| *CHEK1* | Checkpoint kinase 1 | 0.77 |
| *DDR2* | Discoidin domain receptor tyrosine kinase 2 | 1.04 |
| *EGF* | Epidermal growth factor | 1.01 |
| *GPR109B* | Hydroxycarboxylic acid receptor 3 | 1.23 |
| *GPR41* | Free fatty acid receptor 3 | 1.02 |
| *GSK3A* | Glycogen synthase kinase 3 alpha | 0.95 |
| *HSPA12A* | Heat shock protein family A (Hsp70) member 12A | 0.96 |
| *HSPA12B* | Heat shock protein family A (Hsp70) member 12B | 0.99 |
| *HSPA14* | Heat shock protein family A (Hsp70) member 14 | 1.30 |
| *HSPA1A* | Heat shock protein family A (Hsp70) member 1A | 1.49 |
| *HSPA1B* | Heat shock protein 1B | 1.67 |
| *ICAM1* | Intercellular adhesion molecule 1 | 1.02 |
| *ICK* | Intestinal cell kinase | 0.97 |
| *LCK* | LCK proto-oncogene, Src family tyrosine kinase | 1.01 |
| *MFGE8* | Milk fat globule-EGF factor 8 protein | 1.13 |
| *MUC2* | Mucin 2, oligomeric mucus/gel-forming | 1.13 |
| *PTEN* | Phosphatase and tensin homolog | 1.07 |
| *PTPN6* | Protein tyrosine phosphatase, non-receptor type 6 | 0.94 |
| *ROCK1* | Rho associated coiled-coil containing protein kinase 1 | 1.15 |
| *SYN1* | Synapsin I | 1.20 |
| **Second component genes** |  |  |
| *BAK1* | BCL2 antagonist/killer 1 | 1.56 |
| *BAX* | BCL2 associated X, apoptosis regulator | 0.98 |
| *CAMK2B* | Calcium/calmodulin dependent protein kinase II beta | 1.10 |
| *CASP4* | caspase 4 | 1.18 |
| *CCNB1* | Cyclin B1 | 1.14 |
| *CDK7* | Cyclin dependent kinase 7 | 1.44 |
| *CDK8* | Cyclin dependent kinase 8 | 1.01 |
| *CDKN1A* | Cyclin dependent kinase inhibitor 1A | 1.30 |
| *ERBB2* | erb-b2 receptor tyrosine kinase 2 | 1.09 |
| *FOS* | Fos proto-oncogene, AP-1 transcription factor subunit | 1.29 |
| *GPR43* | free fatty acid receptor 2 | 1.07 |
| *GSK3A* | Glycogen synthase kinase 3 alpha | 0.95 |
| *HSPA12B* | Heat shock protein family A (Hsp70) member 12B | 0.99 |
| *HSPA14* | Heat shock protein family A (Hsp70) member 14 | 1.30 |
| *HSPA1A* | Heat shock protein family A (Hsp70) member 1A | 1.49 |
| *HSPA1B* | Heat shock protein 1B | 1.67 |
| *ICAM1* | Intercellular adhesion molecule 1 | 1.02 |
| *ICK* | Intestinal cell kinase | 0.97 |
| *LCK* | LCK proto-oncogene, Src family tyrosine kinase | 1.01 |
| *PTEN* | Phosphatase and tensin homolog | 1.07 |
| *PTPN6* | Protein tyrosine phosphatase, non-receptor type 6 | 0.94 |
| *SYN1* | Synapsin I | 1.20 |

***** Mean values were calculated using normalized count data for the respective breast-fed (BF) or formula-fed (FF) samples.

**Table S6.** List of host immunology and defense-related that form the horizontal (first component) and the vertical (second component) axes in the sPCA plot shown in Figure 3.

| **Genes ID** | **Gene Name** | **Mean BF/Mean FF*** |
| --- | --- | --- |
| **First component genes** | |  |
| *B2M* | Beta-2-microglobulin | 1.46 |
| *BPI* | Bactericidal permeability-increasing protein | 0.89 |
| *BPIL1* | BPI fold-containing family B member 2 | 0.82 |
| *C1QL2* | Complement C1q-like protein 2 | 0.98 |
| *C1QL4* | Complement C1q-like protein 4 | 0.99 |
| *CCL18* | C-C motif chemokine 18 | 0.97 |
| *CCL22* | C-C motif chemokine 22 | 0.97 |
| *CD1C* | T-cell surface glycoprotein CD1c | 0.93 |
| *CD207* | C-type lectin domain family 4 member K | 0.94 |
| *CD7* | T-cell antigen CD7 | 1.04 |
| *CD79A* | B-cell antigen receptor complex-associated protein alpha chain | 0.96 |
| *CNIH2* | Protein cornichon homolog 2 | 0.94 |
| *CYSLTR2* | Cysteinyl leukotriene receptor 2 | 0.97 |
| *DEFB105A* | Beta-defensin 105 | 1.75 |
| *DEFB118* | Beta-defensin 118 | 1.04 |
| *DUOX2* | Dual oxidase 2 | 1.04 |
| *F12* | Coagulation factor XII | 1.17 |
| *F9* | Coagulation factor IX | 0.97 |
| *FCGR2A* | Low affinity immunoglobulin gamma Fc region receptor II-a | 0.85 |
| *FGFR1* | Fibroblast growth factor receptor 1 | 0.95 |
| *HLA-DOB* | HLA class II histocompatibility antigen, DO beta chain | 0.96 |
| *HLA-DQB2* | HLA class II histocompatibility antigen, DQ beta 2 chain | 1.03 |
| *HLA-F* | HLA class I histocompatibility antigen, alpha chain F | 1.30 |
| *HLA-G* | HLA class I histocompatibility antigen, alpha chain G | 1.31 |
| *HSPA1A* | Heat shock 70 kDa protein 1A | 1.49 |
| *ICAM1* | Intercellular adhesion molecule 1 | 1.02 |
| *IER3* | Radiation-inducible immediate-early gene | 1.28 |
| *IFITM2* | Interferon-induced transmembrane protein 2 | 1.16 |
| *IL17RC* | Interleukin-17 receptor C, IL-17 receptor C | 0.95 |
| *IL17RE* | Interleukin-17 receptor E | 0.98 |
| *IL1B* | Interleukin-1 beta | 1.34 |
| *IL4* | Interleukin-4 | 0.95 |
| *IL8* | Interleukin-8 | 1.33 |
| *ITGA3* | Integrin alpha-3 | 0.98 |
| *KLRF1* | Killer cell lectin-like receptor subfamily F member 1 | 0.81 |
| *LGALS3* | Galectin-3 | 1.43 |
| *LGALS4* | Galectin-4 | 1.18 |
| *LRRC8E* | Volume-regulated anion channel subunit LRRC8E | 1.02 |
| *MCFD2* | Multiple coagulation factor deficiency protein 2 | 1.11 |
| *NPTXR* | Neuronal pentraxin receptor | 1.00 |
| *PPIL4* | Peptidyl-prolyl cis-trans isomerase-like 4 | 1.00 |
| *PYGO2* | Pygopus homolog 2 | 1.05 |
| *SRC* | Proto-oncogene tyrosine-protein kinase Src | 1.07 |
| *TBX21* | T-box transcription factor | 0.91 |
| *VANGL2* | Vang-like protein 2 | 0.86 |
| **Second component genes** | | |
| *CCL22* | C-C motif chemokine 22 | 0.97 |
| *CD7* | T-cell antigen CD7 | 1.04 |
| *F12* | Coagulation factor XII | 1.17 |
| *HLA-F* | HLA class I histocompatibility antigen, alpha chain F | 1.30 |
| *IL17RE* | Interleukin-17 receptor E | 0.98 |
| *IL1B* | Interleukin-1 beta | 1.34 |
| *LGALS3* | Galectin-3 | 1.43 |
| *LGALS4* | Galectin-4 | 1.18 |
| *TST* | Thiosulfate sulfurtransferase | 1.28 |
| *SEPP1* | Selenoprotein P | 1.40 |
| *S100A8* | Protein S100-A8 | 1.61 |
| *HSPA8* | Heat shock cognate 71 kDa protein | 1.30 |
| *KLF6* | Krueppel-like factor 6 | 1.22 |
| *CD63* | CD63 antigen | 1.47 |
| *LGALS2* | Galectin-2 | 1.25 |
| *HSPA1B* | Heat shock 70 kDa protein 1B | 1.67 |
| *TYROBP* | TYRO protein tyrosine kinase-binding protein | 1.36 |
| *MICA* | MHC class I polypeptide-related sequence A | 1.24 |
| *LGALS3BP* | Galectin-3-binding protein | 0.98 |
| *MPST* | 3-mercaptopyruvate sulfurtransferase | 1.09 |
| *HSPB1* | Heat shock protein beta-1 | 1.40 |
| *IGBP1* | Immunoglobulin-binding protein 1 | 1.13 |
| *CCL8* | C-C motif chemokine 8 | 0.94 |
| *CASP1* | Caspase-1 | 1.41 |
| *TPT1* | Translationally-controlled tumor protein | 1.39 |
| *DEFA5* | Defensin-5 | 1.09 |
| *IFITM1* | Interferon-induced transmembrane protein 1 | 1.18 |
| *CCL14* | C-C motif chemokine 14 | 1.44 |
| *S100A9* | Protein S100-A9 | 1.25 |
| *NFKBIA* | NF-kappa-B inhibitor alpha | 1.48 |
| *OASL* | 2'-5'-oligoadenylate synthase-like protein | 1.34 |
| *IRF7* | Interferon regulatory factor 7 | 1.37 |
| *CCL4* | C-C motif chemokine 4 | 1.21 |
| *IL1F9* | Interleukin-36 gamma | 0.76 |
| *DDT* | D-dopachrome decarboxylase | 1.31 |
| *CRB3* | Protein crumbs homolog 3 | 1.28 |
| *ARHGAP9* | Rho GTPase-activating protein 9 | 1.21 |
| *GADD45A* | Growth arrest and DNA damage-inducible protein GADD45 alpha | 1.55 |
| *SMAD9* | Mothers against decapentaplegic homolog 9 | 1.10 |

***** Mean values were calculated using normalized count data for the respective breast-fed (BF) or formula-fed (FF) samples.

**Table S7a.** List of immunology and defense host genes that form the horizontal (first component) and the vertical (second component) axes of the sCCA plot depicted in Figure 4a.

| **Genes ID** | **Gene Name** | **Mean BF/Mean FF*** |
| --- | --- | --- |
| **First component genes** | | |
| DEFB106A | Beta-defensin 106 | 1.30 |
| FBXO42 | F-box only protein 42 | 1.10 |
| NR3C1 | Glucocorticoid receptor | 1.06 |
| ITGB5 | Integrin beta-5 | 1.02 |
| LTBP4 | Latent- transforming growth factor-beta-binding protein 4 | 1.02 |
| C1QL4 | Complement C1q-like protein 4 | 0.99 |
| IL22 | Interleukin-22 | 0.99 |
| SLIT3 | Slit homolog 3 protein | 0.99 |
| AHSG | Alpha-2-HS-glycoprotein | 0.98 |
| IFNB1 | Interferon beta | 0.98 |
| LGALS3BP | Galectin-3-binding protein | 0.98 |
| PRG3 | Proteoglycan 3 | 0.98 |
| IL17B | Interleukin-17beta | 0.97 |
| IKBKAP/ELP1 | Elongator complex protein 1 | 0.96 |
| ARHGAP17 | Rho GTPase-activating protein 17 | 0.93 |
| **Second component genes** | |  |
| SERP1 | Stress-associated endoplasmic reticulum protein 1 | 1.21 |
| CEBPB | CCAAT/enhancer-binding protein beta | 1.30 |
| FGF2 | Fibroblast growth factor 2 | 0.91 |
| CLCF1 | Cardiotrophin-like cytokine factor 1 | 0.95 |
| PROZ | Vitamin K-dependent protein Z | 0.86 |
| HRH4 | Histamine H4 receptor | 0.82 |
| CD22 | B-cell receptor CD22 | 0.89 |
| CD3E | T-cell surface glycoprotein CD3 epsilon chain | 0.91 |
| BPIL1/BPIFB2 | BPI fold-containing family B member 2 | 0.82 |
| SPACA3 | Sperm acrosome membrane-associated protein 3 | 0.86 |
| PROCR | Endothelial protein C receptor | 0.89 |
| PTPN22 | Tyrosine-protein phosphatase non-receptor type 22 | 0.83 |

***** Mean values were calculated using normalized count data for the respective breast-fed (BF) or formula-fed (FF) samples.

**Table 7Sb.** List of SEEDLevel2 microbial categories that form the horizontal (first component) and the vertical (second component) axes depicted in Figure 4b.

| **Genes ID** | **Mean BF/Mean FF*** |
| --- | --- |
| **First component SEEDLevel2 Categories** |  |
| RNA Metabolism_Transcription | 0.99 |
| Clustering-Based Subsystems_Recx and Regulatory Cluster | 0.94 |
| **Second component SEEDLevel2 Categories** |  |
| Clustering-based subsystems putative associate of RNA polymerase sigma-54 factor rpoN | 1.34 |
| Clustering-based subsystems related to N-acetylglucosamine utilization | 1.17 |

*Mean values were calculated using normalized count data for the respective breast-fed (BF) or formula-fed (FF) samples.

**Table S8.** List of host barrier function-related that form the horizontal (first component) and the vertical (second component) axes in the sPCA plot shown in Figure 5.

| **Genes ID** | **Gene Name** | **Mean BF/Mean FF*** |
| --- | --- | --- |
| **First component genes** | |  |
| *ACTA1* | Actin, alpha skeletal muscle | 0.96 |
| *ACTA2* | Actin, aortic smooth muscle | 1.05 |
| *CDH2* | Cadherin-2 | 1.02 |
| *CGNL1* | Cingulin-like protein 1 | 1.03 |
| *CLDN4* | Claudin-4 | 1.13 |
| *CTNND1* | Catenin delta-1 | 0.91 |
| *CTNND2* | Catenin delta-2 | 1.04 |
| *CXCL1* | Growth-regulated alpha protein | 0.93 |
| *CXCL2* | C-X-C motif chemokine 2 | 1.16 |
| *F11R* | Junctional adhesion molecule A | 1.05 |
| *IL1B* | Interleukin-1 beta | 1.34 |
| *JAM3* | Junctional adhesion molecule C | 1.09 |
| *MAGI2* | Membrane-associated guanylate kinase, WW and PDZ domain-containing protein 2 | 0.95 |
| *MLLT4* | Afadin | 0.99 |
| *MUC2* | Mucin-2 | 1.13 |
| *MYH2* | Myosin-2 | 1.01 |
| *PVRL1* | Nectin-1 | 1.18 |
| *TNF* | Tumor necrosis factor | 1.01 |
| **Second component genes** | | |
| *ACTG1* | Actin, cytoplasmic 2 | 1.58 |
| *ACTA1* | Actin, alpha skeletal muscle | 0.96 |
| *CDH1* | Fizzy-related protein homolog | 1.15 |
| *CLDN3* | Claudin-3 | 1.15 |
| *CLDN4* | Claudin-4 | 1.13 |
| *CTNNA1* | Catenin alpha-1 | 1.18 |
| *CTNND1* | Catenin delta-1 | 0.91 |
| *CXCL1* | Growth-regulated alpha protein | 0.93 |
| *CXCL2* | C-X-C motif chemokine 2 | 1.16 |
| *F11R* | Junctional adhesion molecule A | 1.05 |
| *IL1B* | Interleukin-1 beta | 1.34 |
| *IKBKG* | NF-kappa-B essential modulator | 1.60 |
| *JAM3* | Junctional adhesion molecule C | 1.09 |
| *MLLT4* | Afadin | 0.99 |
| *PARD3* | Partitioning defective 3 homolog | 1.07 |
| *TNF* | Tumor necrosis factor | 1.01 |

***** Mean values were calculated using normalized count data for the respective breast-fed (BF) or formula-fed (FF) sample.

**Table S9a.** List of barrier function-related host genes that form the horizontal (first component) and the vertical (second component) axes of the sCCA plot depicted in Figure 6a.

| **Genes ID** | **Gene Name** | **Mean BF / Mean FF*** |
| --- | --- | --- |
| **First component genes** |  |  |
| CXCL2 | Chemokine ligand 2 | 1.16 |
| CLDN4 | Claudin 4 | 1.13 |
| ACTA1 | Actin | 0.96 |
| CLDN1 | Claudin 1 | 0.95 |
| **Second component genes** |  |  |
| ACTG1 | Actin, cytoplasmic 2 | 1.58 |
| CTNND1 | Catenin, delta 1 | 0.91 |

*Mean values were calculated using normalized count data for the respective breast-fed (BF) or formula-fed (FF) samples.

**Table S9b.** List of SEEDLevel2 microbial categories that form the horizontal (first component) and the vertical (second component) axes depicted in Figure 6b.

| **Genes ID** | **Mean BF / Mean FF*** | |
| --- | --- | --- |
| **First component SEEDLevel2 Categories** | |  |
| Sulfur Metabolism_Organic sulfur assimilation | | 1.05 |
| Carbohydrates_Clustering-based subsystems | | 1.03 |
| RNA Metabolism_Transcription | | 0.99 |
| Clustering-based subsystems | | 0.98 |
| Cell Division and Cell Cycle_Cell cycle in Prokaryota | | 0.97 |
| Clustering-based subsystems_Protein export | | 0.97 |
| DNA Metabolism_DNA repair | | 0.97 |
| Cell Wall and Capsule | | 0.96 |
| Clustering-based subsystems_Cell Division | | 0.96 |
| Protein Metabolism_Selenoproteins | | 0.94 |
| Respiration_ATP synthases | | 0.94 |
| Protein Metabolism_Lipoproteins | | 0.93 |
| **Second component SEEDLevel2 Categories** | |  |
| Clustering-based subsystems_Putative asociate of RNA polymerase sigma-54 factor rpoN | | 1.34 |
| Clustering-based subsystems_Related to N-acetylglucosamine utilization subsystem | | 1.17 |
| DNA Metabolism | | 1.14 |
| Cell Wall and Capsule_Capsular and extracellular polysacchrides | | 1.07 |
| Virulence_Resistance to antibiotics and toxic compounds | | 1.02 |
| Carbohydrates_Central carbohydrate metabolism | | 1.01 |
| Motility and Chemotaxis_Flagellar motility in Prokaryota | | 0.95 |
| Motility and Chemotaxis | | 0.93 |
| Clustering-based subsystems_Hypothetical in Lysine biosynthetic cluster | | 0.87 |
| Clustering-based subsystems_Hypothetical protein possible functionally linked with Alanyl-tRNA synthetase | | 0.86 |
| Fatty Acids and Lipids_Triacylglycerols | | 0.85 |

*Mean values were calculated using normalized count data for the respective breast-fed (BF) or formula-fed (FF) samples.

**Table S10.** Simulation comparison between the performance of sub-dimensional CCA, sCCA and sPCA.

| **Method** | **n** | **p** | **q** | **p_0_** | **q_0_** | **TPR** | **SE_TPR_** | **FPR** |
| --- | --- | --- | --- | --- | --- | --- | --- | --- |
| sub-dimensional CCA | 12 | 40 | 10 | 10 | 10 | 0.245 | 0.014 | 0.252 |
| sCCA | 12 | 40 | 10 | 10 | 10 | 0.465 | 0.032 | 0.262 |
| sPCA | 12 | 40 | 10 | 10 | 10 | 0.394 | 0.034 | 0.198 |
| sub-dimensional CCA | 12 | 100 | 10 | 10 | 10 | 0.127 | 0.01 | 0.097 |
| sCCA | 12 | 100 | 10 | 10 | 10 | 0.424 | 0.035 | 0.24 |
| sPCA | 12 | 100 | 10 | 10 | 10 | 0.276 | 0.031 | 0.08 |
| sub-dimensional CCA | 12 | 600 | 100 | 10 | 10 | n/a | n/a | n/a |
| sCCA | 12 | 600 | 100 | 10 | 10 | 0.318 | 0.032 | 0.225 |
| sPCA | 12 | 600 | 100 | 10 | 10 | 0.054 | 0.01 | 0.008 |
| sub-dimensional CCA | 12 | 600 | 100 | 10 | 10 | n/a | n/a | n/a |
| sCCA | 100 | 600 | 100 | 10 | 10 | 0.954 | 0.014 | 0.37 |
| sPCA | 100 | 600 | 100 | 10 | 10 | 0.74 | 0.041 | 0.036 |
| sub-dimensional CCA | 100 | 1600 | 100 | 10 | 10 | n/a | n/a | n/a |
| sCCA | 100 | 1600 | 100 | 10 | 10 | 0.933 | 0.005 | 0.434 |
| sPCA | 100 | 1600 | 100 | 10 | 10 | 0.19 | 0.035 | 0.007 |
| sub-dimensional CCA | 100 | 16000 | 100 | 10 | 10 | n/a | n/a | n/a |
| sCCA | 100 | 16000 | 100 | 10 | 10 | 0.937 | 0.015 | 0.296 |
| sPCA | 100 | 16000 | 100 | 10 | 10 | 0.005 | 0.002 | 0.001 |

Abbreviations: FPR, false positive rate; TPR, true positive rate; SE_TPR_, standard error of the TPR. n, p, q, p_0_, and q_0_ represent the sample size, total number of host genes, total number of SEEDLevel2 microbial categories, true number of host genes that are associated with the microbiota, and the true number of microbial categories that are associated with the host transcriptome, respectively. n/a field indicates that the sub-dimensional CCA could not be used in the respective setting.
